# N6-methyladenosine reader YTHDF2 promotes multiple myeloma cell proliferation through EGR1/p21^cip1/waf1^/CDK2-Cyclin E1 axis-mediated cell cycle transition

**DOI:** 10.1038/s41388-023-02675-w

**Published:** 2023-04-03

**Authors:** Rui Liu, Jiyu Miao, Yachun Jia, Guangyao Kong, Fei Hong, Fangmei Li, Meng Zhai, Ru Zhang, Jiaxi Liu, Xuezhu Xu, Ting Wang, Hui Liu, Jinsong Hu, Yun Yang, Aili He

**Affiliations:** 1 Department of Hematology, The Second Affiliated Hospital of Xi’an Jiaotong University, 157, 5th West Road, 710004 Xi’an, Shaanxi China; 2 National-Local Joint Engineering Research Center of Biodiagnostics & Biotherapy, The Second Affiliated Hospital of Xi’an Jiaotong University, Xi’an, 710004 Shaanxi China; 3 Department of Tumor and Immunology in precision medical institute, Xi’an Jiaotong University, Xi’an, China; 4 Department of Cell Biology and Genetics, The Institute of Infection and Immunity, Xi’an Jiaotong University Health Science Center, Xi’an, China

**Keywords:** Myeloma, Cell growth, RNA metabolism

## Abstract

Multiple myeloma (MM) is the second most common hematological malignancy. N6-methyladenosine (m^6^A) is the most abundant RNA modification. YTH domain-containing family protein 2 (YTHDF2) recognizes m^6^A-cotaining RNAs and accelerates degradation to regulate cancer progression. However, the role of YTHDF2 in MM remains unclear. We investigated the expression levels and prognostic role of YTHDF2 in MM, and studied the effect of YTHDF2 on MM proliferation and cell cycle. The results showed that YTHDF2 was highly expressed in MM and was an independent prognostic factor for MM survival. Silencing YTHDF2 suppressed cell proliferation and caused the G_1_/S phase cell cycle arrest. RNA immunoprecipitation (RIP) and m^6^A-RIP (MeRIP) revealed that YTHDF2 accelerated EGR1 mRNA degradation in an m^6^A-dependent manner. Moreover, overexpression of YTHDF2 promoted MM growth via the m^6^A-dependent degradation of EGR1 both in vitro and in vivo. Furthermore, EGR1 suppressed cell proliferation and retarded cell cycle by activating p21^cip1/waf1^ transcription and inhibiting CDK2-cyclinE1. EGR1 knockdown could reverse the inhibited proliferation and cell cycle arrest upon YTHDF2 knockdown. In conclusion, the high expression of YTHDF2 promoted MM cell proliferation via EGR1/p21^cip1/waf1^/CDK2-cyclin E1 axis-mediated cell cycle transition, highlighting the potential of YTHDF2 as an effective prognostic biomarker and a promising therapeutic target for MM.

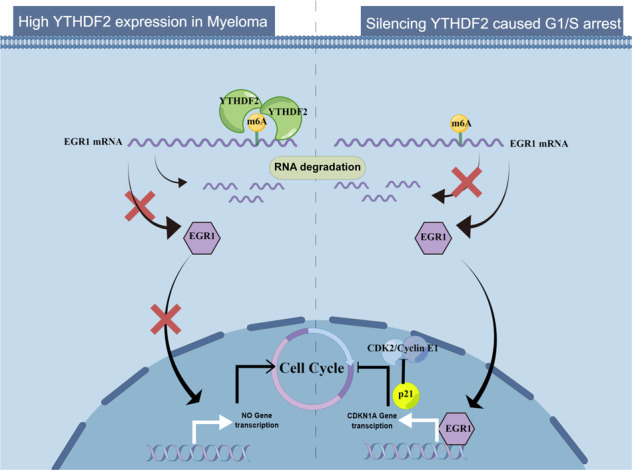

## Introduction

Multiple myeloma (MM) is the second most common hematological malignancy characterized by the clonal proliferation of malignant plasm cells in the bone marrow [1]. Initiation of MM is characterized by monoclonal gammopathy of unknown significance (MGUS) and smoldering MM (SMM), finally developing into the extramedullary MM (EMM) and plasma cell leukemia (PCL). Treatment of MM involves the use of proteasome inhibitor-based regimen, and is supplemented with immunotherapy, including monoclonal antibodies and chimeric antigen receptor-engineered T cells [2]. However, MM remains incurable with high mortality rate. Therefore, it is urgent to understand the pathogenesis of MM and explore novel therapeutic targets.

N6-methyladenosine (m^6^A) is the most abundant RNA modification among more than 160 types of chemical post-transcriptional modifications [3–5]. M^6^A sites mainly occur at the consensus motif of RRACH (R = G/A, H = A/C/U) in 3’untranslated regions (3’UTR), around stop codons, within internal long exons and precursor mRNAs [6, 7]. Notably, the process of m^6^A RNA methylation is reversible: m^6^A is installed by “writers”, removed by “erasers”, and recognized by “readers”. Methyltransferase-like 3 (METTL3) and METTL14, key components of methyltransferase complex, cooperating with adapter proteins, such as WT1-associated protein (WTAP), conduct the methyl transfer. On the other hand, fat mass and obesity-associated protein (FTO) and α-ketoglutarate-dependent dioxygenase homolog 5 (ALKBH5) are RNA demethylases responsible for the removal of m^6^A modification [8]. Additionally, readers can recognize specific m^6^A sites and regulate RNA splicing, miRNA processing, RNA export, degradation and translation [9, 10].

YTH domain-containing family protein 2 (YTHDF2) is the first identified m^6^A reader. YTHDF2 can recognize m^6^A-cotaining RNA and accelerate the degradation process to regulate many biological processes, such as viral infection, stem cell development and cancer progression [11–13]. Paris et al. [14] demonstrated that YTHDF2 played an essential role in the initiation and development of leukemia stem cells. Moreover, YTHDF2 was shown to promote epithelial-mesenchymal transition and AKT phosphorylation to support prostate cancer progression [15]. However, the role of YTHDF2 in MM progression remains largely unclear. In this study, we observed the increased expression of YTHDF2 in MM, and showed that YTHDF2 was an independent prognostic factor for overall survival (OS) in MM. Mechanistically, this study revealed that YTHDF2 promoted MM cell proliferation through the EGR1/p21^cip1/waf1^/CDK2-Cyclin E1 axis-mediated cell cycle transition. These findings suggested that YTHDF2 could be a potential biomarker and promising therapeutic target in MM.

## Methods and Materials

### Cell lines and cell culture

Human myeloma cell lines MM.1S, RPMI-8226, NCI-H929 were purchased from the American Type Culture Collection (USA), OPM2 and U266 were obtained from Professor Jinsong Hu of Xi’an Jiaotong University Health Science Center (Xian, Shaanxi, China). MM cell lines were maintained in RPMI-1640 (Hyclone, Logan, UT, USA) with 10% fetal bovine serum (FBS) (Biological Industries, Kibbutz Beit Haemek, Israel), penicillin (10,000 U/L, BioSharp, Hefei, China) and streptomycin (100 mg/L, BioSharp, Hefei, China). All cells were incubated at 37 °C in a humidified atmosphere with 5% CO_2_.

### Patients

Bone marrow specimens were obtained from newly diagnosed MM at the Department of Hematology, the Second Affiliated Hospital of Xi’an Jiaotong University, from 2018 to 2022. Peripheral blood-derived mononuclear cells from healthy doners were used as control. The clinical samples used in this study to identify the expression of YTHDF2 were supervised and granted by the Ethics Committee of the Second Affiliated Hospital of Xi’an Jiaotong University (2015186).

### RNA immunoprecipitation (RIP) assay

RIP assay was performed using the BersinBio^TM^ RNA Immunoprecipitation Kit (BersinBio, Guangzhou, China) according to the manufacturers’ instruction. Polysome lysis buffer was used to lyse cells, and the cell lysate was divided into anti-YTHDF2, anti-IgG (1 mg/ml, Cell Signaling Technology) and input samples. The cell lysates were incubated with pre-conjugated protein A/G magnetic beads with 5 μg of specific antibodies overnight at 4 °C. After washing, the lysates were digested with Proteinase K for 1 h at 55 °C, and the RNA bound to immunoprecipitated proteins was purified by phenol: chloroform: isoamyl alcohol (25:24:1). Realtime-qPCR was performed to measure the target RNA expression levels. All primer sequences are shown in Supplementary Table 1.

### m^6^A-RNA immunoprecipitation (MeRIP) assay

MeRIP assay was performed using the BersinBio^TM^ MeRIP Kit (BersinBio, Guangzhou, China) according to the manufacturers’ instruction. Total RNA was extracted using TRIzol reagent. Then, the RNA was sheared into about 300-nucleotide fragments. Next, RNA was incubated with 5 μg anti-m^6^A antibody (68055-1-Ig, Proteintech) or IgG for 2 h at 4 °C. Protein A/G magnetic beads were mixed with the antibody-treated RNA in IP buffer for 2 h at 4 °C. Then, the bound RNAs were washed and eluted with Proteinase K and elution buffer for 1 h at 55 °C. Finally, RNA bound to immunoprecipitated proteins was purified by phenol: chloroform: isoamyl alcohol (25:24:1). Realtime-qPCR was performed to measure the methylated RNA expression levels.

### RNA stability assay

Cells were treated with Actinomycin D (5 μg/ml, MCE, USA) for 0, 10, 20, 40, 60 min or longer. Total RNA was then extracted by TRIzol. The target RNA was measured by Realtime-qPCR. 18 s rRNA was used as the endogenous standard control for mRNA normalization. The mRNA degradation rate K_dacay_ and RNA lifetime (t_1/2_) can be calculated using the equations [16]: Nt/N0 = e ^– Kdecay*t^, t_1/2_ = ln2/K_decay_.

### Animal experiment

Animal experiment was supervised and granted by the Ethic Committee of Xi’an Jiaotong University Health Science Center (2022-1497). Male BALB/c nude mice (4 weeks old) were use in this study. The mice were fed in specific pathogen-free facilities. RPMI-8226 cells transfected with negative control (NC) or shYTHDF2 or LV-oeYTHDF2 were injected into the left and right flank of mice. Tumor volume was recorded every 3 days, and calculated by the formula: (width^2^ × length × 0.5). Mice were sacrificed 4 weeks after injection or when the tumor diameter was more than 15 mm. Tumors were sent for further immunohistochemistry (IHC) staining as previously described [17].

### Chromatin immunoprecipitation (ChIP) assay

ChIP assay was performed by the ChIP assay Kit (Beyotime, China) according to the manufacturer’s instruction. Briefly, one million MM cells were cross-linked with 1% formaldehyde for 10 min at 37 °C. Subsequently, 0.125 M glycine was added to terminate the reaction, and cells were lysed on ice by lysis buffer. Chromatin DNA was sonicated to obtained 200-1000 bp fragments, which was followed by incubation with anti-EGR1 antibody or IgG at 4 °C overnight. EGR1-bound DNA fragments were precipitated by Protein A + G Agarose/Salmon sperm DNA. After de-crosslink and purification, qPCR and agarose gel electrophoresis were employed to analyze the precipitated DNA. The EGR1-binding sequence of *p21*^*cip1/waf1*^ promoter was predicted by JASPAR database (https://jaspar.genereg.net/) [18].

### Statistical analysis

Three dependent biological replicates were used in each experiment. Mann-Whitney test and Student’s t test were used to compare the difference in two groups as appropriate. One-way ANOVA followed by Bonferroni post hoc comparison were employed to compare the difference of more than two subgroups. *P* < 0.05 was considered statistically significant if not specified. All statistical tests were two-sided.

## Results

### YTHDF2 is highly expressed in MM patients

**Fig. 1:**
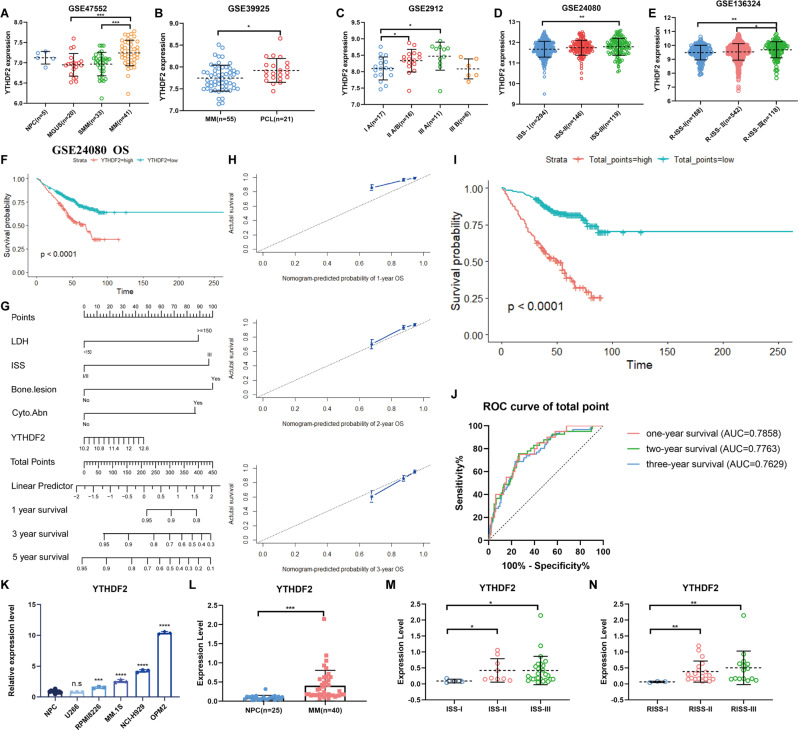
YTHDF2 is highly expressed in MM, and is correlated prognosis. **A–E** the YTHDF2 expression in MM progression: normal plasma cell (NPC), MGUS, SMM, MM (**A**); PCL (**B**); DSS (**C**), ISS (**D**) and R-ISS stages (**E**). **F** Kaplan-Meier survival analysis showing the OS of MM patients having the low YTHDF2 expression (blue) and high YTHDF2 expression (red). **G** Nomogram predicting 1-, 3-, 5-year survival of MM patients. (**H**) Calibration plots of the nomogram for 1-year, 2-year and 3-year OS. (**I**) Kaplan-Meier survival analysis for total points of Nomogram. **J** ROC curve for the nomogram prognosis system. **K** The expression of YTHDF2 in MM cell lines and normal plasm cells by RT-qPCR. **L–N** The expression of YTHDF2 in normal plasma cells and MM patients grouped by ISS and R-ISS stages. Data are mean ± SD values. *P < 0.05, **P < 0.01, ***P < 0.001, ****P < 0.0001, n.s. not significant.

To explore the association between YTHDF2 expression and MM, we performed a comprehensive analysis of the development of MM. The results revealed significantly higher expression of YTHDF2 in MM than in both MGUS (*P* = 0.0008) and SMM (*P* = 0.0002) (Fig. 1A). Moreover, the expression of YTHDF2 was higher in PCL than in MM (*P* = 0.0210, Fig. 1B). Furthermore, higher Durie-Salmon system (DSS), International Staging System (ISS) and Revised-ISS (R-ISS) stages were associated with increased expression of YTHDF2 in MM patients (Fig. 1C–E). There was also a significant increase in the expression of YTHDF2 in relapsed MM patients (Supplementary Fig. 1A–C) and those with bortezomib (BTZ) resistance (Supplementary Fig. 1D, E). Overall, these findings showed that YTHDF2 had an oncogenic role in MM, and that was positively correlated with advanced-stage MM, recurrence and resistance to bortezomib.

### The expression of YTHDF2 is an independent factor for MM survival

The results further showed that the OS, event-free survival (EFS) and progression-free survival (PFS) of MM patients with higher levels of YTHDF2 expression was shorter than that of patients with low YTHDF2 expression (Fig. 1F, Supplementary Fig. 1F–J). In addition, univariate and multivariate Cox regression analyses revealed that the expression level of YTHDF2 was an independent prognostic factor for MM survival (*P* = 0.02, Table 1). We further combined YTHDF2 expression with other independent prognostic factors and constructed a nomogram to calculate the total points (Fig. 1G). The calibration curves illustrated great agreement between the predicted and actual 1-, 2- and 3- year survival probabilities (Fig. 1H). Notably, MM patients with higher total points showed significantly poorer OS than those with lower total points (*P* < 0.0001, Fig. 1I). The area under ROC curve (AUC) of the total points for 1-, 2- and 3- year survival probabilities was 0.7858 (95%CI: 0.6934 to 0.8773, *P* < 0.0001), 0.7763 (95%CI: 0.7013 to 0.8513, *P* < 0.0001), and 0.7629 (95%CI: 0.6982 to 0.8275, *P* < 0.0001), respectively (Fig. 1J). ROC analysis and decision curve analysis (DCA) further showed that the inclusion of YTHDF2 could improve the evaluation accuracy of MM prognosis (Supplementary Fig. 1K–N).

**Table 1:** Univariate and multivariate Cox regression analyses for OS of MM patients.

variables	univariate analysis		multivariate analysis				
	HR (95%CI)	*P* value	beta	SE	Wald	HR (95%CI)	*P* value
Age, years	0.953 (0.593–1.531)	0.842					
Gender	1.103 (0.733–1.660)	0.638					
Race	1.145 (0.639–2.052)	0.649					
LDH(U/l)	2.564 (1.685–3.903)	**<0.0001**	0.72	0.219	10.762	2.054 (1.336–3.157)	**0.001**
ALB(g/l)	0.533 (0.339–0.838)	**0.006**	−0.234	0.247	0.894	0.792 (0.488–1.285)	0.344
HGB(g/dl)	0.546 (0.310–0.961)	**0.036**	−0.031	0.312	0.01	0.792 (0.488–1.285)	0.921
ISS stage	2.531 (1.705–3.759)	**<0.0001**	0.593	0.23	6.626	1.810 (1.152–2.843)	**0.010**
BMPC	2.118 (1.404–3.197)	**<0.0001**	0.418	0.228	3.36	1.519 (0.971–2.376)	0.067
cytogenetic abnormalities	2.662 (1.788–3.961)	**<0.0001**	0.638	0.212	9.093	1.893 (1.250–2.865)	**0.003**
bone lesions	2.246 (1.374–3.670)	**0.001**	0.858	0.255	11.34	2.359 (1.431–3.886)	**0.001**
YTHDF2	1.849 (1.233–2.773)	**0.003**	0.491	0.211	5.421	1.634 (1.081–2.471)	**0.020**

*HR* hazed ratio, *SE* standard error, *CI* credibility interval, *LDH* lactate dehydrogenase, *ALB* albumin, *HGB* hemoglobin, *BMPC* bone marrow plasm cell.

Next, we examined YTHDF2 expression in both MM cell lines and patients. The findings revealed that YTHDF2 was highly expressed in MM cell lines and patients compared to normal plasm cells (Fig. 1K, L). In addition, there was increased expression of YTHDF2 in MM patients with more advanced ISS and R-ISS stage (Fig. 1M, N). Collectively, the results suggested that YTHDF2 could be a potential biomarker for predicting MM prognosis, and the combination of YTHDF2 with clinical features could effectively improve the accuracy of prognosis evaluation.

### Silencing YTHDF2 inhibits MM cell proliferation, and causes G_1_/S phase cell cycle arrest

**Fig. 2:**
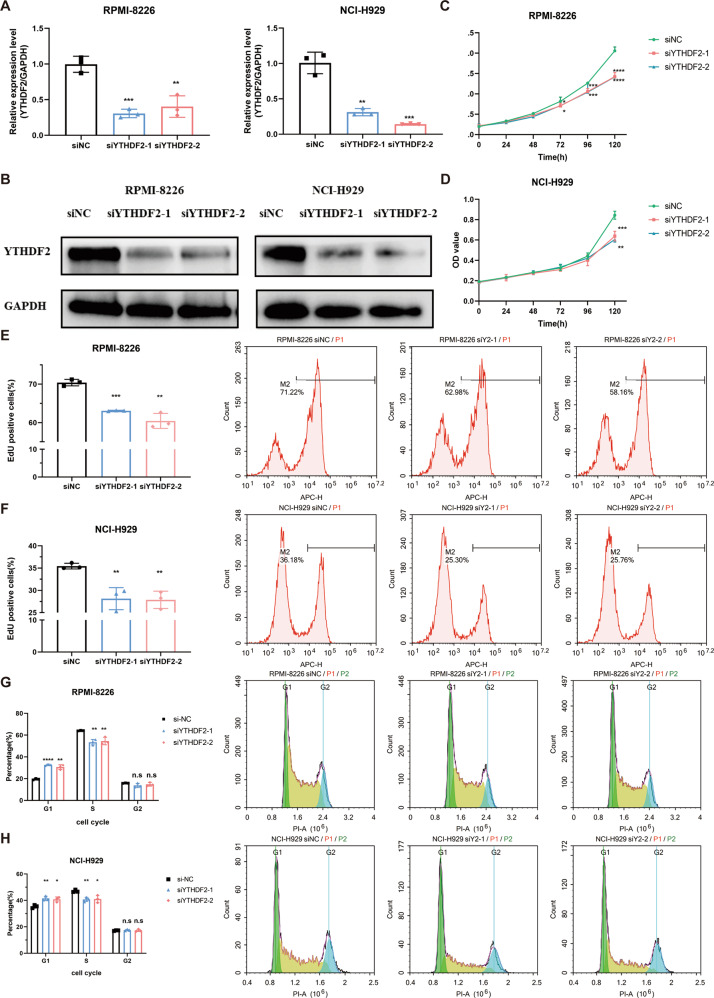
Silencing YTHDF2 inhibits MM cell proliferation, and causes G_1_/S phase cell cycle arrest. **A**, **B** Validation of YTHDF2 knockdown at both mRNA and protein levels in RPMI-8226 and NCI-H929 MM cell lines. **C**, **D** Cell viability was detected by CCK8 assays in RPMI-8226 and NCI-H929 cells transfected with siNC and siYTHDF2. **E**, **F** Cell proliferation was measured by EdU assay in RPMI-8226 and NCI-H929 cells transfected with siRNAs. **G**, **H** Cell cycle was detected by PI-staining in RPMI-8226 and NCI-H929 cells transfected with siNC and siYTHDF2. Data are mean ± SD values. *P < 0.05, **P < 0.01, ***P < 0.001, ****P < 0.0001, n.s. not significant.

To further explore the role of YTHDF2 in MM, we downregulated YTHDF2 expression in MM cell lines RPMI-8226 and NCI-H929 using specific siRNAs (Fig. 2A, B). The results showed the knockdown of YTHDF2 had no significant effect on the expression levels of the other m^6^A regulators, METTL3, FTO, ALKBH5, YTHDF1, and YTHDF3 (*P* > 0.05, Supplementary Fig. 2A–D). We observed that the knockdown of YTHDF2 significantly inhibited cell viability (Fig. 2C, D) and cell proliferation (Fig. 2E, F). Cell cycle analysis further revealed that the downregulation of YTHDF2 in RPMI-8226 and NCI-H929 resulted in the increase of G_1_ phase cells and reduction of S phase cells, while no significant difference was observed in the G_2_ phase, suggesting possible G_1_/S phase arrest (Fig. 2G, H).

To reinforce our findings, MM cell lines were transfected with lentivirus vectors carrying YTHDF2-shRNA and scrambled shRNA. The findings similarly showed that downregulation of YTHDF2 significantly suppressed cell proliferation and caused cell cycle arrest (Supplementary Fig. 2E–H).

### YTHDF2 enhances the degradation of EGR1 mRNA in an m^6^A-dependent manner

**Fig. 3:**
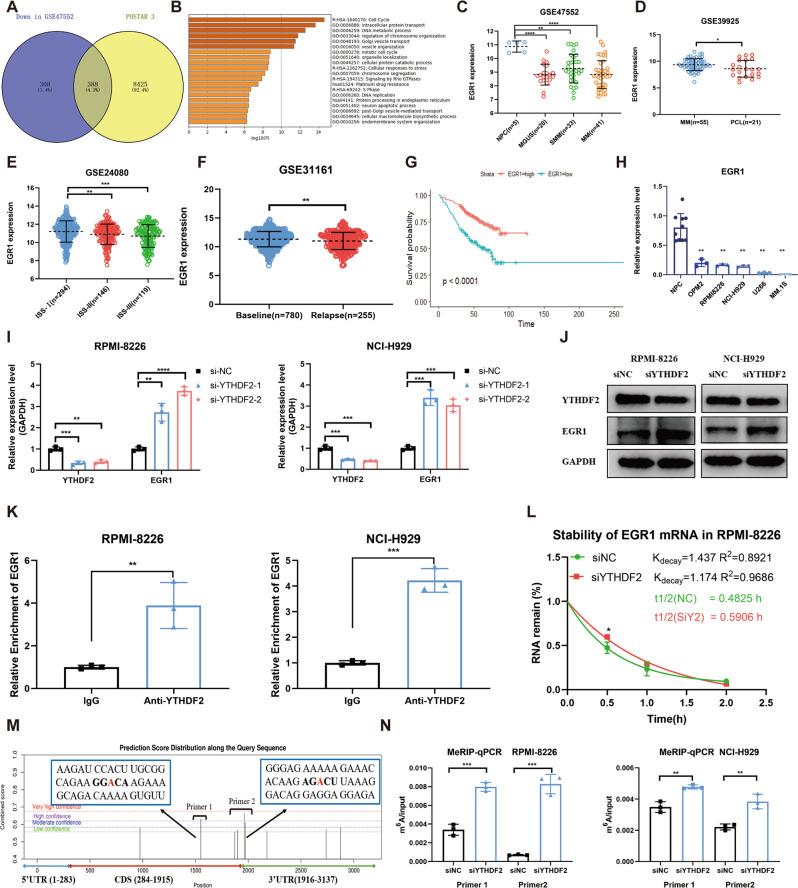
YTHDF2 enhances the degradation of EGR1 mRNA in an m^6^A-dependent manner. **A** The Venn plot showed 388 overlapped genes between significantly downregulated genes in MM from GSE47552 and potential RNA targets of YTHDF2 from POSTAR database. **B** The enrichment analysis was conducted by Metascape. **C–F** The expression of EGR1 in the development of MM: NPC, MGUS, SMM, MM (**C**); PCL (**D**); ISS stage (**E**) and relapsed MM patients (**F**). **G** Kaplan-Meier survival analysis showing the OS for MM patients having low EGR1 expression (blue) and high EGR1 expression (red) in the GSE24080 dataset. **H** The expression of EGR1 in MM cell lines and normal plasm cells. **I**, **J** The mRNA and protein levels of EGR1 upon YTHDF2 knockdown in MM cell lines RPMI-8226 and NCI-H929. **K** RNA immunoprecipitation assays of EGR1 transcripts in YTHDF2-bound mRNAs in RPMI-8226 and NCI-H929 cells. **L** RNA stability analysis of EGR1 after actinomycin D treatment in RPMI-8226 cells transfected with siNC and siYTHDF2. **M** The putative m^6^A sites of EGR1 mRNA were predicted by POSTAR3. **N** Methylated RNA immunoprecipitation of EGR1 transcripts in RPMI-8226 and NCI-H929 transfected with siNC and siYTHDF2. Data are mean ± SD values. *P < 0.05, **P < 0.01, ***P < 0.001, ****P < 0.0001, n.s. not significant.

Given that YTHDF2 could promote the degradation of m^6^a-containing transcripts, we analyzed the significantly downregulated genes in MM patients relative to healthy individuals based on the GSE47552 dataset. We combined these downregulated genes with PAR-CLIP-seq results of YTHDF2 from POSTAR3 online database (http://lulab.life.tsinghua.edu.cn/postar/) [19], finally obtaining 388 genes (Fig. 3A, Supplementary Table 2). Next, Gene Ontology and pathway enrichment analyses were conducted using the online Metascape tool (https://metascape.org/gp) [20], which showed that YTHDF2 was most involved in the cell cycle (Fig. 3B, Supplementary Table 3). Among these genes related to cell cycle, early growth response factor 1 (EGR1) was the most downregulated one (fold change = −4.14, Supplementary Table 2), hence selected for further validation. Furthermore, we found a significant negative correlation between the expression of YTHDF2 and EGR1 (Supplementary Fig. 3A–C). Compared with normal plasm cells, the expression of EGR1 was lower in MGUS, SMM and MM (Fig. 3C). In addition, there was a significant decrease in the expression of EGR1 in PCL and MM patients with a higher ISS stage as well as in relapsed MM patients (Fig. 3D–F). It was also shown that MM patients with low expression of EGR1 had remarkably poorer OS (Fig. 3G). Moreover, we confirmed the downregulation of EGR1 in MM cell lines compared with normal plasm cells (Fig. 3H).

Further, the ability of YTHDF2 to regulate the expression of EGR1 in an m^6^A-dependent manner was investigated. The findings showed that knockdown of YTHDF2 significantly upregulated the expression of EGR1 (Fig. 3I, J). In addition, RIP-qPCR verified the interaction between the YTHDF2 protein and EGR1 mRNA (Fig. 3K). The half-life of EGR1 mRNA was also significantly prolonged in YTHDF2-downregulated MM cells (28.95 min versus 35.44 min, *P* = 0.0377), suggesting that YTHDF2 could decrease the stability of EGR1 mRNA (Fig. 3L). Then, several potential m^6^A sites for EGR1 mRNA were discovered using SRAMP [21] (http://www.cuilab.cn/sramp/, Fig. 3M), and MeRIP-qPCR was performed. Knockdown of YTHDF2 significantly increased m^6^A modification of EGR1 mRNA in MM cells (Fig. 3N). Taken together, these results suggested that EGR1 mRNA was a direct target of YTHDF2, and YTHDF2 could accelerate EGR1 mRNA degradation in an m^6^A-dependent manner in MM.

### EGR1 inhibits cell proliferation and causes cell cycle arrest by transcriptionally regulating p21^cip1/waf1^

**Fig. 4:**
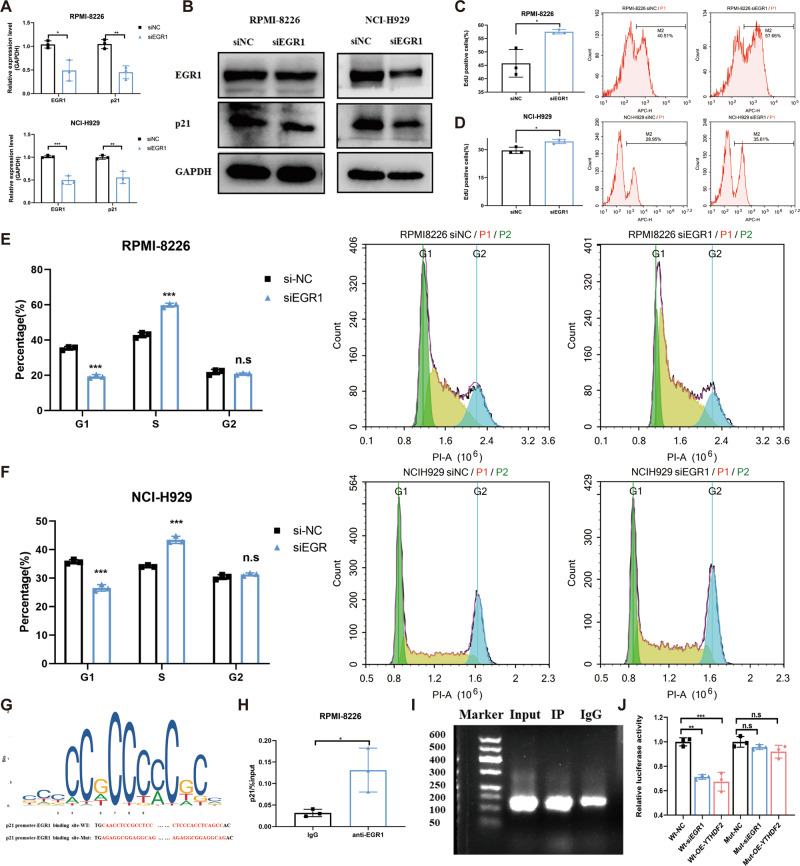
EGR1 inhibits cell proliferation and causes cell cycle arrest by transcriptionally regulating p21^cip1/waf1^. **A**, **B** The mRNA and protein levels of EGR1 and p21^cip1/waf1^ upon EGR1 knockdown in MM cell lines RPMI-8226 and NCI-H929. **C**, **D** Cell proliferation was measured by EdU assay in RPMI-8226 and NCI-H929 cells transfected with siNC or siEGR1. (**E**, **F**) Cell cycle was detected by PI staining in RPMI-8226 and NCI-H929 cells transfected with siNC and siEGR1. **G** The EGR1-binding motif of p21 promoter (above) was predicted by JASPAR database. **H**, **I** ChIP assay was analyzed by RT-qPCR and agarose gel electrophoresis. **J** Luciferase reporter assays of wt- and mut-p21^cip1/waf1^ promoter sequences following EGR1 knockdown or YTHDF2 overexpression. Data are mean ± SD values. *P < 0.05, **P < 0.01, ***P < 0.001, ****P < 0.0001, n.s. not significant.

To further validate the role of EGR1 in MM, EGR1 was silenced in MM cell lines (Fig. 4A, B). The EdU assay showed that silencing EGR1 significantly enhanced cell proliferation (Fig. 4C, D). Moreover, cell cycle analysis revealed that the downregulation of EGR1 caused an increase in S phase cells and a decrease in G_1_ phase cells, suggesting the promotion of the G_1_/S phase (Fig. 4E, F).

The cyclin-dependent kinase inhibitor 1 A (CDKN1A), also named p21^cip1/waf1^, is a well-studied tumor suppressor, known for mediating cell cycle arrest [22]. Previous studies have shown that EGR1, as a transcriptional factor, can promote the activation of p21^cip1/waf1^ in glioma [23, 24], breast cancer [25], melanoma [26] and gastric cancer [27]. In addition, a positive correlation between EGR1 and p21^cip1/waf1^ expression was found in MM (Supplementary Fig. 3D, E). Therefore, we hypothesized that EGR1 could suppress the proliferation of MM cells and cause cell cycle arrest by activating p21^cip1/waf1^ transcription. Expectedly, the knockdown of EGR1 significantly decreased the expression of p21^cip1/waf1^ (Fig. 4A, B). To further assess whether EGR1 was involved in the transcriptional activation of p21^cip1/waf1^ in MM cells, we predicted the potential EGR1-binding motif on the p21^cip1/waf1^ promoter using JASPAR (Fig. 4G). The ChIP-qPCR assay confirmed the interaction between EGR1 and p21^cip1/waf1^ promoter (Fig. 4H, I). In addition, we constructed luciferase reporter plasmids with wild type (wt) or mutant (mut) sequences, and found that luciferase activity in the wt-luciferase reporter was significantly decreased upon both the knockdown of EGR1 and the overexpression of YTHDF2. However, no significant difference was observed in the mut-luciferase reporter (Fig. 4J). Collectively, these findings suggested that EGR1 could suppress the proliferation of MM cells and retard cell cycle by transcriptionally activating p21^cip1/waf1^.

### Silencing YTHDF2 suppresses cell proliferation and cell cycle via the EGR1/ p21^cip1/waf1^/CDK2-Cyclin E1 axis

**Fig. 5:**
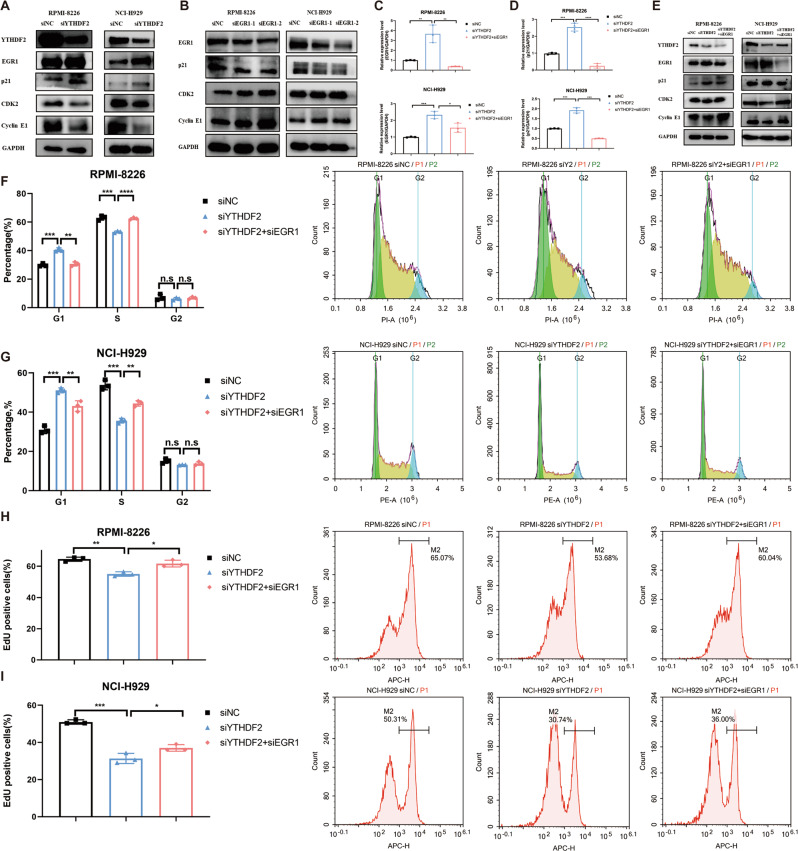
Silencing YTHDF2 suppresses cell proliferation via the EGR1/ p21^cip1/waf1^/CDK2-Cyclin E1 axis. **A**, **B** The protein levels of the YTHDF2/EGR1/ p21^cip1/waf1^/CDK2-Cyclin E1 axis upon YTHDF2 knockdown (**A**) and EGR1 knockdown (**B**) in MM cell lines RPMI-8226 and NCI-H929. **C**, **D** Realtime-qPCR results of the EGR1 (**C**) and p21^cip1/waf1^ (**D**) mRNA expression upon silencing both YTHDF2 and EGR1 in RPMI-8226 and NCI-H929. **E** The protein levels of the YTHDF2/EGR1/ p21^cip1/waf1^/CDK2-Cyclin E1 axis upon silencing both YTHDF2 and EGR1 in RPMI-8226 and NCI-H929. **F**, **G** Cell cycle was detected by PI staining in RPMI-8226 and NCI-H929 cells simultaneously transfected with siYTHDF2 and siEGR1. **H**, **I** Cell proliferation was measured by EdU assay in RPMI-8226 and NCI-H929 cells simultaneously transfected with siYTHDF2 and siEGR1. Data are mean ± SD values. *P < 0.05, **P < 0.01, ***P < 0.001, ****P < 0.0001, n.s. not significant.

Previous research demonstrated that cyclin-dependent kinase 2 (CDK2)-Cyclin E1 was involved in p21^cip1/waf1^-mediated G_1_/S cell cycle transition [22]. Therefore, we speculated that YTHDF2 could regulate cell proliferation and cell cycle via the EGR1/p21^cip1/waf1^/CDK2-Cyclin E1 axis in MM. As expected, there was an increase in p21^cip1/waf1^ expression, while CDK2 and Cyclin E1 expression decreased following YTHDF2 knockdown in MM cell lines (Fig. 5A). Conversely, knockdown of EGR1 caused the downregulation of p21^cip1/waf1^, but resulted in the upregulation of CDK2 and cyclin E1 (Fig. 5B).

To further investigate the YTHDF2/EGR1/p21^cip1/waf1^/CDK2-Cyclin E1 axis in MM, we simultaneously silenced YTHDF2 and EGR1 in MM cell lines. Knockdown of EGR1 not only reversed the increased expression of EGR1 and p21^cip1/waf1^, but also the decreased expression of CDK2 and Cyclin E1 caused by YTHDF2 silencing (Fig. 5C–E). Additionally, cell cycle analysis showed that EGR1 knockdown significantly reversed the G_1_/S phase arrest induced by YTHDF2 knockdown (Fig. 5F, G). The EdU assays also showed that silencing EGR1 significantly enhanced cell proliferation in YTHDF2-knockdown MM cells (Fig. 5H, I). Overall, these results demonstrated that YTHDF2 could promote cell proliferation and G_1_/S transition via the EGR1/p21^cip1/waf1^/CDK2-Cyclin E1 axis in MM.

### Knockdown of YTHDF2 suppresses tumor growth in MM xenograft models

**Fig. 6:**
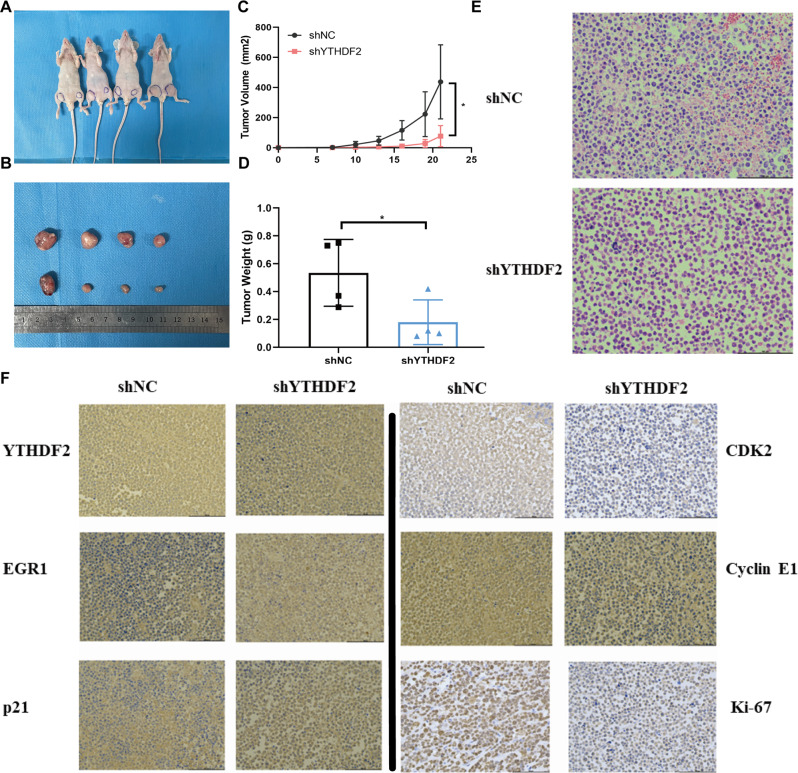
Knockdown of YTHDF2 suppresses tumor growth in MM xenograft models. **A** RPMI-8226 cells transfected with shNC or shYTHDF2 were injected in the left or right flanks of BALB/c nude mice (*n* = 4). **B** Mice were sacrificed when tumor diameter was more than 15 mm, xenograft tumors were completely dissected. **C** Tumor volume was recorded every 3 days and calculated by the formula: (width^2^ × length ×0.5). (**D**) Tumor weight was recorded after dissection. **E** Representative hematoxylin-eosin (H&E) staining. **F** Representative IHC staining of YTHDF2, EGR1, p21^cip1/waf1^, CDK2, Cyclin E1 and Ki-67 in tumors from negative control and YTHDF2-downregulated nude mice. Data are mean ± SD values. *P < 0.05.

To assess the effect of YTHDF2 on MM proliferation in vivo, we subcutaneously injected RPMI-8226 cells transfected with shNC or shYTHDF2 into nude mice. Generally, tumors from shYTHDF2-transfected RPMI-8226 cells were visually smaller than those from shNC-transfected RPMI-8226 cells (Fig. 6A, B). Knockdown of YTHDF2 significantly decreased both tumor volume (*P* = 0.0304, Fig. 6C) and tumor weight (*P* = 0.0496, Fig. 6D). IHC results confirmed the decreased expression of YTHDF2, CDK2, Cyclin E1, and Ki-67, and the increased expression of EGR1 and p21^cip1/waf1^ in YTHDF2-downreuglated xenografts (Fig. 6E, F; Supplementary Fig. 2I). Taken together, these data confirmed the oncogenic role of YTHDF2 in promoting MM proliferation via the EGR1/p21^cip1/waf1^/CDK2-Cyclin E1 axis in vivo.

### Overexpression of YTHDF2 promotes MM growth in vitro and in vivo

**Fig. 7:**
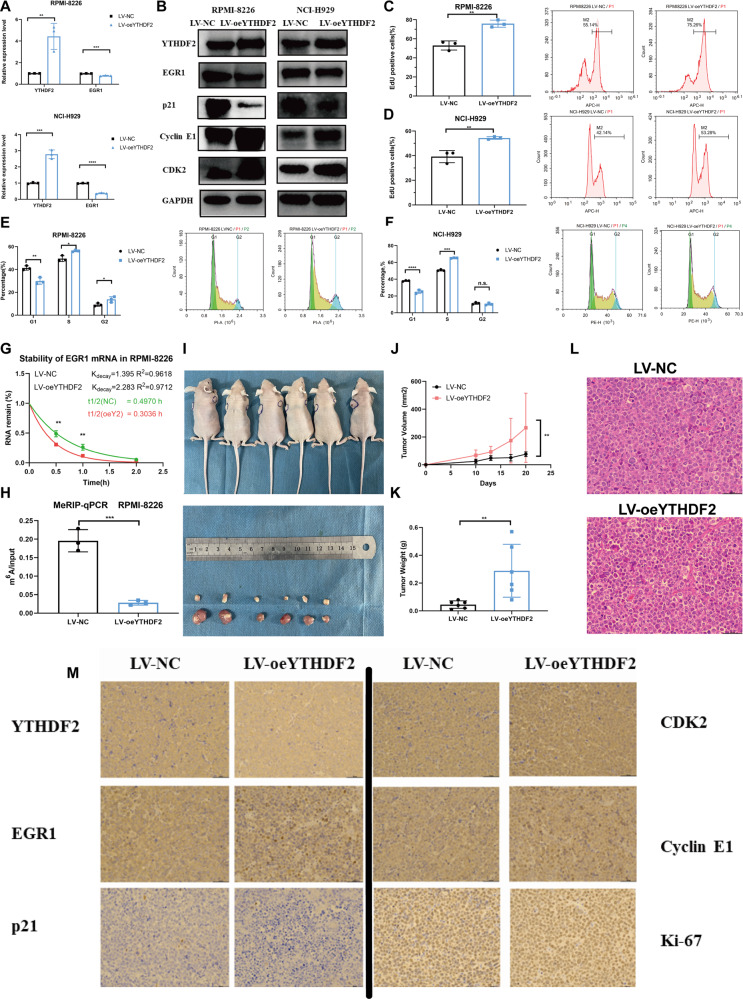
Overexpression of YTHDF2 enhances MM growth via the m^6^A-dependent degradation of EGR1 in vitro and in vivo. **A**, **B** The expression of the YTHDF2/EGR1/ p21^cip1/waf1^/CDK2-Cyclin E1 in RPMI-8226 and NCI-H929 transfected with LV-NC and LV-oeYTHDF2. **C**, **D** Cell proliferation was measured by EdU assay in RPMI-8226 and NCI-H929 transfected with LV-NC and LV-oeYTHDF2. **E**, **F** Cell cycle was detected by PI-staining in RPMI-8226 and NCI-H929 transfected with LV-NC and LV-oeYTHDF2. **G** RNA stability analysis of EGR1 after actinomycin D treatment upon YTHDF2 overexpression. **H** Methylated RNA immunoprecipitation of EGR1 transcripts in YTHDF2-upregulated MM cells. **I** RPMI-8226 cells transfected with LV-NC or LV-oeYTHDF2 were injected in the left or right flanks of BALB/c nude mice (*n* = 6). Mice were sacrificed when tumor diameter was more than 15 mm, xenograft tumors were completely dissected. **J** Tumor volume was recorded every 3 days and calculated by the formula: (width^2^ × length ×0.5). (**K**) Tumor weight was recorded after dissection. **L** Representative H&E staining in tumors from LV-NC (above) and LV-oeYTHDF2 (below) mice. **M** Representative IHC staining of YTHDF2, EGR1, p21^cip1/waf1^, CDK2, Cyclin E1 and Ki-67 in tumors from LV-NC and LV-oeYTHDF2 nude mice. Data are mean ± SD values. *P < 0.05, **P < 0.01, ***P < 0.001, ****P < 0.0001, n.s. not significant.

The findings further showed that the overexpression of YTHDF2 by lentiviral transduction (LV-oeYTHDF2) significantly reduced the expression of EGR1 and p21, but enhanced the expression of CDK2 and Cyclin E1 (Fig. 7A, B). In addition, the upregulation of YTHDF2 significantly promoted cell proliferation and the progression of cell cycle (Fig. 7C–F). The half-life of EGR1 mRNA was significantly shortened in MM cells overexpressing YTHDF2 (29.82 min versus 18.216 min; Fig. 7G). The MeRIP-qPCR also showed that overexpression of YTHDF2 significantly decreased the m^6^A modification of EGR1 mRNA (Fig. 7H).

Furthermore, we subcutaneously injected RPMI-8226 cells transfected with LV-NC and LV-oeYTHDF2 into nude mice, and observed that tumors from the LV-oeYTHDF2 group were bigger than those from LV-NC group (Fig. 7I). Additionally, the overexpression of YTHDF2 significantly increased both tumor volume (*P* = 0.0063, Fig. 7J) and tumor weight (*P* = 0.0076, Fig. 7K). IHC results confirmed the decreased expression of EGR1 and p21^cip1/waf1^, and increased expression of YTHDF2, CDK2, Cyclin E1, and Ki-67 in LV-oeYTHDF2 xenografts compared with the LV-NC group (Fig. 7L, M, Supplementary Fig. 2J).

## Discussion

MM is a highly heterogeneous and genetically complicated hematological malignancy. According to existing evidence, MM initiation and progression is mainly dependent on a series of chromosomal and genetic alterations, such as IgH translocation, hyperdiploidy, amplification of the 1q chromosome, KRAS and TP53 mutations [28]. Accurate risk stratification and novel therapeutic regimens have significantly improved the survival of MM patients. However, the considerable clonal heterogeneity of MM makes it challenging to implement individualized and targeted therapy [29]. In the recent years, epigenetic abnormalities, such as RNA modifications along with DNA and histone methylation, have been demonstrated to contribute to MM heterogeneity [30]. Notably, m^6^A is the most common form of RNA modification, and recent studies have shown that m^6^A regulators are dysregulated in multiple cancers. Nevertheless, the role of m^6^A modification in MM oncogenesis remains unknown. In this study, the expression of YTHDF2 in MM patients was shown to be significantly higher than that in healthy individuals. Additionally, the knockdown of YTHDF2 significantly inhibited the proliferation of MM cells, caused cell cycle G_1_/S cycle arrest in vitro, and suppressed tumor growth in vivo. However, the overexpression of YTHDF2 reversed these effects. Mechanistically, YTHDF2 recognized and accelerated the degradation of EGR1 mRNA in an m^6^A-dependent manner. Moreover, without the transcriptional activation of EGR1, the reduced p21^cip1/waf1^ expression reversed its inhibitory effect on CDK2-Cyclin E1 and promoted G_1_/S phase transition to facilitate MM growth.

YTHDF2 is an important m^6^A reader in regulating RNA degradation. In the human YTHDF2-YTH domain, the aromatic cage composed of W486, W432, and W491 of the hydrophobic pocket was shown to recognize and trap m^6^A sites [31, 32]. Then, YTHDF2 could induce RNA decay through the deadenylation-dependent [33] and endonuclease-mediated pathways [34]. Since YTHDF2 functions to regulate gene expression post-transcriptionally, accumulating evidence suggests that YTHDF2 is involved in oncogenesis. For instance, Li et al. [35] revealed that YTHDF2 was highly expressed in lung adenocarcinoma and promoted tumor growth by degrading AXIN1 mRNA and activating the Wnt/beta-catenin signaling pathway. However, the biofunction of YTHDF2 is heterogeneous in cancers, with some studies demonstrating its tumor-inhibiting function. In hepatocellular carcinoma, Zhong et al. [36] revealed that HIF-1α could suppress YTHDF2 expression and activate the ERK/MEK signaling pathway to promote tumor growth by stabilizing EGFR mRNA. However, little research emphasizes the role of YTHDF2 in MM. Recently, Hua et al. [37] reported increased expression of YTHDF2 in MM, which was associated with poor prognosis. They demonstrated that YTHDF2 promoted MM proliferation, consistent with our results, further strengthening the carcinogenic role of YTHDF2 in MM. They also confirmed that YTHDF2 promoted MM progression by degrading STAT5 mRNA and activating the MP2K2/p-ERK signaling pathway. In the present study, the findings revealed that the expression of YTHDF2 was positively correlated with MM stage, recurrence and resistance. Moreover, high expression of YTHDF2 was shown to be an independent and adverse factor for the OS of MM patients. In addition, we showed that silencing YTHDF2 could not only inhibit MM proliferation but also cause the G_1_/S phase arrest in cell cycle. Similarly, Huang et al. [38] reported that YTHDF2 was upregulated in intrahepatic cholangiocarcinoma, and its knockdown resulted in the suppressed proliferation and G_0_/G_1_ cell cycle arrest by promoting CDKN1B (p27) degradation, further validating the role of YTHDF2 in cell cycle regulation.

To further demonstrate the potential mechanism through which YTHDF2 promoted MM proliferation and cell cycle regulation, we analyzed RNA-seq and CLIP-seq data from public databases and identified EGR1 as a potential target for YTHDF2. RIP-qPCR and MeRIP-qPCR results demonstrated that YTHDF2 could interact with the EGR1 mRNA to accelerate its degradation in an m^6^A-dependent manner. Concordantly, Liao et al. [38] showed that EGR1 mRNA was the target for METTL3 and YTHDF3. The EGR1 mRNA underwent m^6^A modifications through the action of METTL3, and YTHDF3 enhanced its RNA stability to upregulate the EGR1/Snail signaling pathway and support cancer metastasis in esophageal squamous cell carcinoma.

The zinc finger transcription factor EGR1 can regulate cell life and death. It consists of a DNA-binding domain, a transcriptional activation domain and an inhibitory domain [39]. However, the role of EGR1 varies in different cancers. As the name suggests, EGR1 can be activated by many growth and inflammatory factors via the MAPK signaling pathway to promote cell proliferation [40]. For example, Grotegut et al. [41] reported that the hepatocyte growth factor could induce cell migration and invasion via the MAPK/EGR1/Snail signaling pathway in liver cancer. In addition, Fahmy et al. [42] showed that EGR1 was crucial for supporting fibroblast growth factor-dependent tumor angiogenesis and tumor growth in breast cancer. EGR1 can also exert its pro-apoptotic and tumor-inhibiting function by transcriptionally activating tumor suppressor genes, such as *TP53*, *p21*^*cip1/waf1*^, *PTEN* and *JUN* [23, 43, 44]. Virolle et al. [43] demonstrated that EGR1 directly transactivated *PTEN* to enhance radiation or chemotherapy-induced apoptosis, and the loss of EGR1 conferred cancer cells with radiation and drug resistance. In MM, mutation of *EGR1* gene was observed in both MM and MGUS [45, 46]. Moreover, Chen et al. [47] reported that EGR1 expression was decreased in MM compared to MGUS and SMM, and low expression of EGR1 was highly associated with poor prognosis, consistent with our results. They further showed that EGR1 was the direct target for JUN, and was critical in bortezomib-induced apoptosis. In this study, we noted a decrease in the levels of EGR1 during the process of MM development. Moreover, knockdown of EGR1 promoted MM cell proliferation and cell cycle transition by decreasing p21^cip1/waf1^ expression and increasing CDK2-Cyclin E1 expression. Additionally, knockdown of EGR1 significantly reversed the inhibited proliferation and G_1_/S phase arrest induced by silencing YTHDF2.

The underlying mechanism contributing to the high expression of YTHDF2 in MM appears to be complex and remains unclear. Transcriptionally, the hypoxia-induced HIF1α/2α signaling pathway can negatively regulate YTHDF2 expression to favor tumor growth in liver cancer [48, 49]. On the other hand, hypoxia was reported to positively regulate the expression of YTHDF2 in lung cancer [50]. At the post-translational level, FBW7 was shown to be involved in ubiquitin-proteasome degradation of YTHDF2 protein in ovarian cancer [51]. In addition, the EGFR/Src/ERK cascade could positively mediate YTHDF2 expressing by stabilizing the YTHDF2 protein through phosphorylation of its serine 39 and threonine 381 [16]. Yang et al. recently reported that the interaction between MM cells and bone marrow adipocytes could promoted the release of adipocyte-derived exocomal LncRNA through the action of METTL17A-mediated m^6^A methylation [52], which further exacerbated MM therapeutic resistance, suggesting that the cellular interaction of MM cells in the bone marrow microenvironment can influence cellular m^6^A levels.

In conclusion, our study demonstrated that YTHDF2 was an independent prognostic factor in MM that could promote tumor growth and cell cycle transition via the EGR1/p21^cip1/waf1^/CDK2-Cyclin E1 axis, highlighting that YTHDF2 could be used as a potential biomarker for predicting prognosis and a promising therapeutic target in MM.

## Supplementary information

Supplementary information

Supplementary materials_original WB

## Data Availability

The datasets used in this article were obtain from the public GEO database.
