# Supplementary material for: N6-methyladenosine reader YTHDF2 promotes multiple myeloma cell proliferation through EGR1/p21cip1/waf1/CDK2-Cyclin E1 axis-mediated cell cycle transition
Source: Oncogene. 2023 Apr 3;42(20):1607–19. doi: 10.1038/s41388-023-02675-w (PMC10181929; doi:10.1038/s41388-023-02675-w)
Supplement: Supplementary file 1 — Supplementary information [file 41388_2023_2675_MOESM1_ESM.docx]

**Supplementary Methods and Materials**

1. **Transient transfection with small interfering RNAs (siRNAs)**

Scramble siRNA, YTHDF2 siRNAs and EGR1 siRNAs were designed by Ribobio (Guangzhou, China) and GenePharma (Suzhou, China). SiRNAs were transfected into cells using the RFect siRNA/miRNA Transfection Reagent (Baidai biotechnology, Changzhou, China) according to the manufacturer’s instruction. The siRNA sense sequences were as follows: siYTHDF2-1: GACCAAGAATGGCATTGCA; siYTHDF2-2: GCACAGAAGTTGCAAGCAA; siEGR1-1: CCCGGTTACTACCTCTTATTT; siEGR1-2: GTGACTGTTTGGCTTATAATT.

1. **Lentivirus vector transduction**

Lentivirus vectors GV493 expressing YTHDF2-shRNA (target sequence: TAGCGGGTCCATTACTAGTAA), oeYTHDF2 and scrambled shRNA were purchased from Genechem Co.Ltd. (Shanghai, China). Transfection was performed as the manufacturer’s instruction. Briefly, 5*10^4^ cells per well were seeded into a 24-well plate and transfected with the indicated lentivirus using HitransG P. Infected cells were selected using 1μg/ml puromycin over 1 week, and the transfection efficiency was determined by flow cytometry, RT-qPCR and Western blot assays.

1. **Western blot**

Cells were dissociated in RIPA buffer (POINEER Biotechnology, Xi’an, China) containing PMSF and protease inhibitors cocktail (NCM Biotech, Suzhou, Xi’an) for 15 min on ice. The cell lysates were centrifuged at 4 ℃ for 15 min at 14000g. The supernatant was collected to measure protein concentration by BCA kit (ShareBio, Shanghai, China). After boiling the protein with SDS loading buffer, the protein samples were separate via SDS-polyacrylamide gel electrophoresis and transferred onto the PVDF membranes. The membranes were blocked by 5% non-fat milk dissolved in TBS-T for 1 h and were then incubated with primary antibodies at 4 ℃ overnight. After washing the membranes by TBS-T for three times, we incubated PVDF membranes with secondary antibodies for 1 h. Signals were observed using an ECL Western blotting detection kit (ShareBio, Shanghai, China). The images were developed by an imaging system (BIO-RAD, ChemiDoc MP, USA). The antibody used in this study were as follows: anti-YTHDF2 (#71283, 1:1000 dilution, Cell Signaling Technology); anti-EGR1 (55117-1-AP, 1:1000 dilution, Proteintech) anti-Cyclin E1 (11554-1-AP, 1:1000 dilution, Proteintech), anti-CDK2 (10122-1-AP, 1:2000 dilution, Proteintech), anti-p21^cip1/waf1^ (10355-1-AP, 1:1000 dilution, Proteintech), anti-GAPDH (ET1601-4, 1:7000 dilution, HUABIO).

1. **RNA isolation and real-time PCR (RT-qPCR)**

Total RNA was extracted using TRIzol reagent, chloroform and isopropanol, and RNA deposit was washed by 75% ethanol. RNA concentration was determined by Thermo Scientific NanoDrop^TM^ One/Onec. cDNA was synthesized using a Primescript RT master mix with Oligo dT primers and random primers in accordance with manufacturer's protocol (CWBIO, China). Then, the qRT-PCR was performed by SYBR Premix Ex Taq™ II (CWBIO, China) and StepOne Software v2.1 according to manufacturer's instructions. Primers were designed and synthesized by Tsingke (Shanghai, China). All primer sequences are shown in **Supplementary Table 1**. 2^-ΔΔCt^ value was used to calculated the expression level of each gene. GAPDH was used as the endogenous standard control for mRNA normalization.

1. **Cell proliferation assays**

Cell proliferation ability was measured by Cell Counting Kit-8 (CCK8) assay and EdU staining assay. The CCK8 assay was to culture 3000 to 5000 cells per well into a 96-well plate for 5 consecutive days with siRNAs transfection. The cell culture medium and CCK8 (Beyotime, Shanghai, China) were mixed with a 10:1 ratio to incubate for 2 h, and then measured the 450 nm absorbance by a microplate reader. EdU staining assay was performed by the BeyoClick^TM^ EdU cell proliferation Kit with Alexa Fluor 647 (Beyotime, Shanghai, China) according to the instruction. Briefly, 2*10^5^ cells per well were cultured in a 12-well plate for siRNAs transfection. After 48 h, the cells were incubated with 10 μM EdU buffer at 37 ℃ for 2h. After centrifuging to collect cells, cells were washed with PBS and fixed with 4% paraformaldehyde for 15 min, then permeabilized with Triton X-100 for 10 min. Then, cells were incubated with 200 μL of click reaction solution for 30 min. The EdU-positive cells were then observed by flow cytometry (ACEA, NovoCyte, USA).

1. **Cell cycle assay**

2*10^5^ cells per well were cultured in a 12-well plate for siRNAs transfection. After centrifuging to collect cells, cells were washed by PBS and fixed with 70% cold ethanol at 4 ℃ overnight for immobilization. After removing ethanol, cells were washed with cold PBS. Then cells were incubated with a mixture of 200 μl propidium iodide (PI) and 50 μL RNase A (Beyotime, Shanghai, China) at room temperature for 30 min in dark. Cell cycle was analyzed by flow cytometry.

1. **Animal experiments**

Animal experiments were supervised and granted by the Ethic Committee of Xi’an Jiaotong University Health Science Center (2022-1497). Male BALB/c nude mice (4 weeks old) were use in this study. The mice were fed in specific pathogen-free facilities. RPMI-8226 cells transfected with negative control or oeYTHDF2 or shYTHDF2 were injected into the left and right flank of mice. Tumor volume was recorded every 3 days and calculated by the formula: (width^2^ * length * 0.5). Mice were sacrificed 4 weeks after injection or when the tumor diameter was more than 15 mm. Tumors were sent for further immunohistochemistry (IHC) staining as previously described. The antibody used in IHC were as follows: anti-YTHDF2 (1:50 dilution); anti-EGR1 (1:200 dilution) anti-Cyclin E1 (1:400 dilution,), anti-CDK2 (1:100 dilution), anti- p21^cip1/waf1^ (1:50 dilution), anti-Ki-67 (27309-1-AP, 1:2000 dilution, Proteintech).

1. **Luciferase reporter gene assay**

Wild type or mutant sequences of p21^cip1/waf1^ promoter were inserted into pGL3-luciferase reporter plasmids. Cells were seeded in a 12-well plate and were transfected with 1.6 μg plasmids. After 48 h, the luciferase intensity was detected by the Luciferase Reporter Gene Assay Kit (Yeasen Biotechnology, Shanghai, China) according to the manufacturer’s instruction.

1. **Bioinformatics analysis**

We obtained the RNA-seq data and clinical data from the GEO database (<https://www.ncbi.nlm.nih.gov/geo>). GSE47552 and GSE39925 datasets were used to compare gene expression in different stages of plasma cell dyscrasia. GSE2912 with Durie-Salmon staging (DSS) information, GSE24080 with the International Staging System (ISS) information and GSE136324 with R-ISS information were used to compare gene expression in different MM stages. GSE9782 and GSE31161 were used to analysis the association of YTHDF2 expression with relapse and drug resistance. GSE4452, GSE57317 and GSE13591 with survival date were used to perform survival analysis. We divided MM patients into high and low YTHDF2 expression and low YTHDF2 expression group upon the optimal cutoff value via “Survminer” package (version 0.4.9; <https://rpkgs.datanovia.com/survminer/index.html>), then Kaplan-Meier survival analysis with log-rank test was used to evaluate the survival rate by the “SURVIVAL” package (version 3.2-7; <https://github.com/therneau/survival>). Univariate and multivariate Cox regression analysis were employed to identify whether these factors were independent for MM prognosis. Briefly, variables with *P*-value<0.05 in univariate analysis were eligible for multivariate Cox regression analysis with forward likelihood ration (LR) method. Then, the nomogram based on independent prognostic factors was constructed using the “rms” package (version 6.2-0; <https://hbiostat.org/R/rms/>). The predictive performance of the nomogram was evaluated with calibration curves and ROC curves. LIMMA package was used to analyze differentially expressed genes between MM and healthy samples (version 3.46.0; <http://bioinf.wehi.edu.au/limma/>) from the R Studio (version 1.4.1103; <https://rstudio.com/>).

**Supplementary Figures**


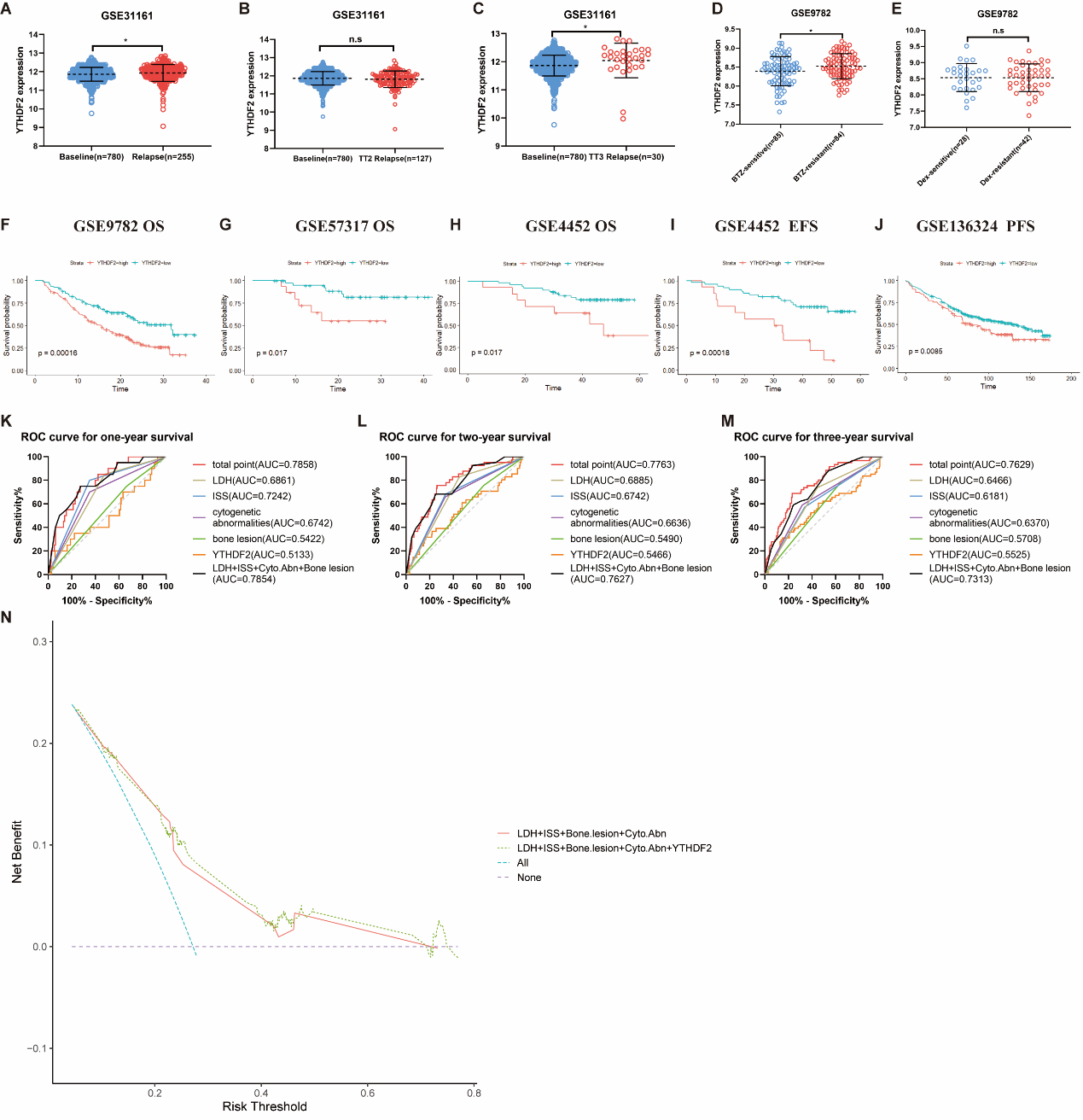


**Supplementary Figures 1. (A-C)** The expression of YTHDF2 in total, TT2 and TT3 relapsed MM patients in the GSE31161 dataset. **(D&E)** The expression of YTHDF2 in MM patients with or without BTZ resistance and DEX resistance in the GSE9782 dataset. **(F-J)** Kaplan-Meier survival analysis showing the OS (**F-H**), EFS (**I**) and PFS **(J)** of MM patients having the low YTHDF2 expression (blue) and high YTHDF2 expression (red). (**K-N)** The inclusion of YTHDF2 for the evaluation of MM prognosis could improve the evaluation accuracy by ROC analysis (**K-M**) and DCA (**N**).


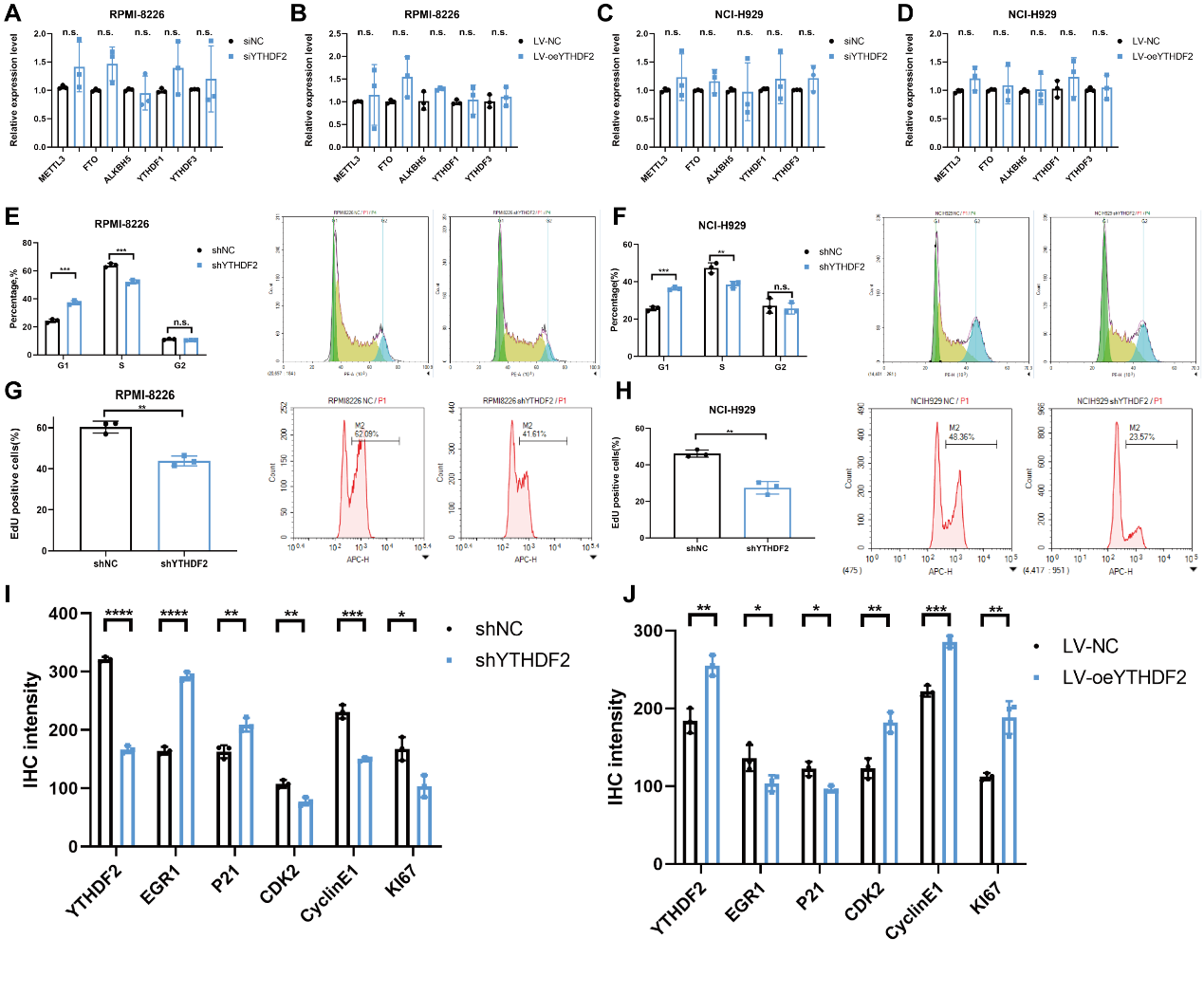


**Supplementary Figures 2. (A-D)** RT-qPCR results showed that knockdown and overexpression of YTHDF2 had no effect on other m^6^A regulators in RPMI-8226 and NCI-H929 MM cell lines. **(E, F)** Cell cycle was detected by PI-staining in RPMI-8226 and NCI-H929 cells transfected with shNC and shYTHDF2. (**G, H**) Cell proliferation was measured by EdU assay in RPMI-8226 and NCI-H929 cells transfected with shNC and shYTHDF2. **(I, J)** Quantification IHC results. Data are mean ± SD values. ** p<0.01, *** p<0.001, n.s. not significant.


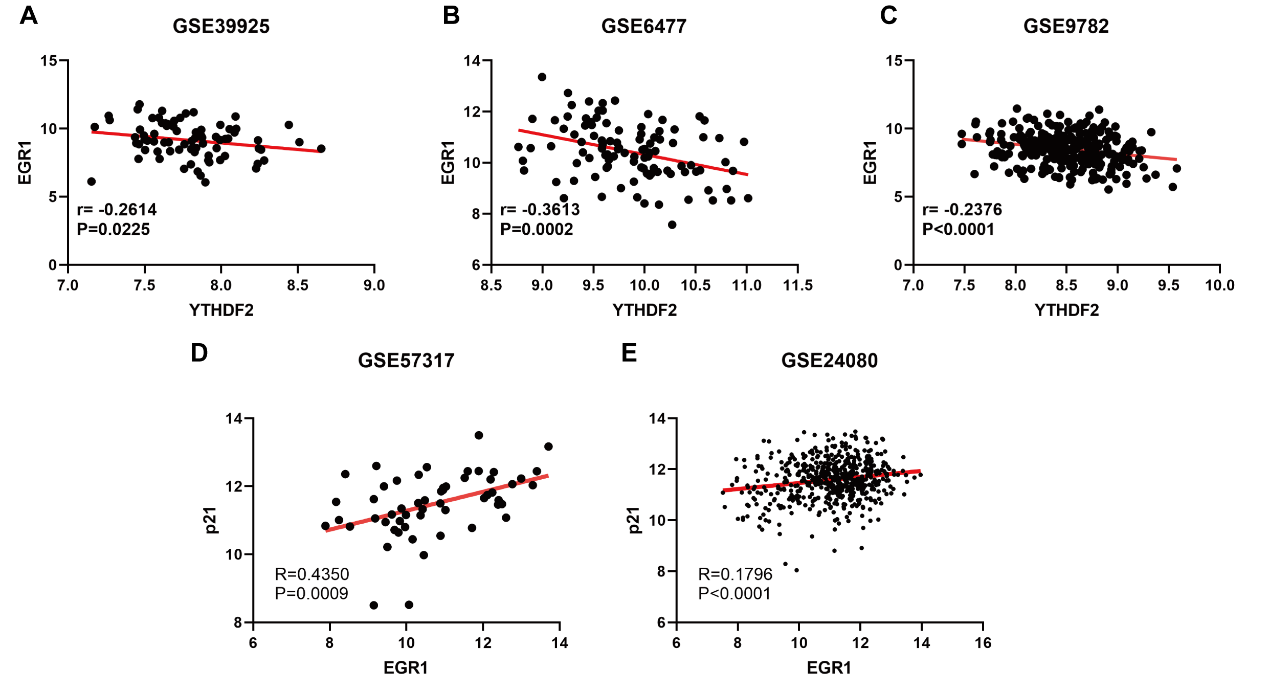


**Supplementary Figures 3. (A-C)** correlation between YTHDF2 expression and EGR1 expression; **(D, E)** correlation between EGR1 expression and p21 expression.

**Supplementary Tables**

**Supplementary Table 1. Primer sequences of RT-qPCR**

| Primers | Sequences (5'-3') |
| --- | --- |
| ALKBH5-F | CGGCGAAGGCTACACTTACG |
| ALKBH5-R | CCACCAGCTTTTGGATCACCA |
| YTHDF1-F | GCCCACAGCTATAACCCTAAA |
| YTHDF1-R | GTGGATGTCGTCCTCAGAATAG |
| YTHDF3-F | TCAGAGTAACAGCTATCCACCA |
| YTHDF3-R | GGTTGTCAGATATGGCATAGGCT |
| YTHDF2-F | TAGCCAACTGCGACACATTC |
| YTHDF2-R | CACGACCTTGACGTTCCTTT |
| GAPDH F | GAAGGTGAAGGTCGGAGTC |
| GAPDH R | GAAGATGGTGATGGGATTTC |
| EGR1-F | GGTCAGTGGCCTAGTGAGC |
| EGR1-R | GTGCCGCTGAGTAAATGGGA |
| 18s rRNA-F | CAGCCACCCGAGATTGAGCA |
| 18s rRNA-R | TAGTAGCGACGGGCGGTGTG |
| METTL3-F | CAAGCTGCACTTCAGACGAA |
| METTL3-R | GCTTGGCGTGTGGTCTTT |
| FTO-F | GCTGCTTATTTCGGGACCTG |
| FTO-R | AGCCTGGATTACCAATGAGGA |
| p21^cip1/waf1-^F | GCCCGTGAGCGATGGAAC |
| p21cip1/waf1-R | CCCCGTGGGAAGGTAGAGC |
| EGR1-m^6^a primer 1-F | AGGAGCGATGAACGCAAGAG |
| EGR1-m6a primer 1-R | GCCACAACACTTTTGTCTGCT |
| EGR1-m6a primer 2-F | CAGGGCTTTCGGACATGACA |
| EGR1-m6a primer 2-R | GAACCCTCCTCTCCTATGGC |
| p21^cip1/waf1^-promoter-F | TCTGTCACCCAGGCTGGAGT |
| p21^cip1/waf1^-promoter-R | GGTGGTGGGCACCTGTAGTC |

**Supplementary Table 2. Differentially expressed analysis of the overlapped genes**

| Gene symbol | logFC | AveExpr | t | P.Value | adj.P.Val | B |
| --- | --- | --- | --- | --- | --- | --- |
| TRIP11 | -2.45647 | 5.643558 | -6.71819 | 1.21E-09 | 3.01E-07 | 11.75118 |
| ZNF675 | -2.45454 | 4.554667 | -5.8071 | 7.86E-08 | 8.79E-06 | 7.767765 |
| CTSH | -2.41459 | 7.24019 | -4.38634 | 0.000029 | 0.000797 | 2.167164 |
| ASXL2 | -2.38598 | 5.966107 | -9.08263 | 1.18E-14 | 2.11E-11 | 22.81068 |
| RB1 | -2.35839 | 7.807537 | -5.51507 | 2.83E-07 | 0.000023 | 6.546976 |
| MINPP1 | -2.25409 | 6.467364 | -5.32862 | 6.32E-07 | 0.0000419 | 5.784407 |
| SEL1L3 | -2.21569 | 9.013428 | -5.075 | 1.84E-06 | 0.000096 | 4.770134 |
| GTF2A1 | -2.1841 | 7.320226 | -9.60091 | 8.83E-16 | 2.12E-12 | 25.28921 |
| GOLPH3L | -2.18403 | 7.11563 | -5.20325 | 1.07E-06 | 0.0000634 | 5.279602 |
| SYNRG | -2.0518 | 6.98971 | -8.78995 | 5.06E-14 | 6.93E-11 | 21.41351 |
| EGR1 | -2.04958 | 9.069576 | -4.54393 | 0.0000158 | 0.0005 | 2.741742 |
| CD81 | -2.02815 | 7.360219 | -5.25147 | 8.77E-07 | 0.0000533 | 5.472998 |
| ORC3 | -2.01873 | 6.5404 | -7.38109 | 5.15E-11 | 2.45E-08 | 14.77674 |
| MANEA | -2.01175 | 8.299631 | -4.76887 | 6.45E-06 | 0.000254 | 3.584225 |
| EPC1 | -1.97605 | 8.128885 | -6.76994 | 9.52E-10 | 2.52E-07 | 11.98409 |
| ROCK2 | -1.9662 | 6.258652 | -7.47172 | 3.33E-11 | 1.99E-08 | 15.19664 |
| SAMHD1 | -1.94279 | 7.147101 | -4.06618 | 0.0000966 | 0.001969 | 1.042103 |
| CABLES1 | -1.93521 | 5.069139 | -7.98529 | 2.71E-12 | 2.26E-09 | 17.59794 |
| PREPL | -1.88759 | 6.789854 | -4.41963 | 0.0000255 | 0.00072 | 2.287426 |
| ITGA6 | -1.85949 | 8.152136 | -3.84129 | 0.000217 | 0.003578 | 0.287844 |
| SLC46A3 | -1.83484 | 5.670896 | -4.94445 | 3.16E-06 | 0.000144 | 4.259082 |
| OSBPL3 | -1.83159 | 5.771044 | -5.17977 | 1.19E-06 | 0.0000682 | 5.185785 |
| SETDB2 | -1.83102 | 5.388348 | -6.19726 | 1.36E-08 | 2.09E-06 | 9.44413 |
| CD9 | -1.81526 | 8.265865 | -2.73752 | 0.007351 | 0.043372 | -2.92405 |
| IRF2 | -1.80982 | 7.243449 | -6.15656 | 1.63E-08 | 2.41E-06 | 9.267034 |
| TRMT1L | -1.8067 | 5.40318 | -5.09077 | 1.72E-06 | 0.0000912 | 4.832402 |
| PDK1 | -1.80087 | 9.43157 | -5.05736 | 1.98E-06 | 0.000101 | 4.700631 |
| LRIF1 | -1.7979 | 5.186132 | -5.17399 | 1.22E-06 | 0.0000695 | 5.162736 |
| KRCC1 | -1.79485 | 4.83363 | -5.65773 | 1.52E-07 | 0.0000149 | 7.139446 |
| ZNF322 | -1.76527 | 5.350712 | -6.56834 | 2.45E-09 | 5.33E-07 | 11.08035 |
| SEC23IP | -1.76055 | 6.999961 | -7.36414 | 5.59E-11 | 2.55E-08 | 14.69834 |
| FAM69A | -1.7544 | 6.841856 | -4.22129 | 0.0000543 | 0.001284 | 1.57988 |
| SRP54 | -1.74873 | 8.066365 | -5.9892 | 3.48E-08 | 4.48E-06 | 8.544153 |
| VCAN | -1.74561 | 7.095924 | -3.34348 | 0.001173 | 0.012264 | -1.26683 |
| FNDC3A | -1.74474 | 8.503709 | -5.17163 | 1.23E-06 | 0.0000697 | 5.15335 |
| SRBD1 | -1.72951 | 6.159762 | -6.55083 | 2.66E-09 | 5.56E-07 | 11.00229 |
| USO1 | -1.71145 | 8.760754 | -4.36492 | 0.0000315 | 0.000853 | 2.090098 |
| PCMTD1 | -1.70807 | 8.707181 | -4.29732 | 0.0000408 | 0.001034 | 1.848541 |
| LARP4B | -1.67178 | 6.389491 | -5.72535 | 1.13E-07 | 0.0000119 | 7.422933 |
| NHLRC2 | -1.6699 | 5.693369 | -6.8859 | 5.51E-10 | 1.66E-07 | 12.5082 |
| DENND4C | -1.66886 | 5.742724 | -4.75126 | 6.92E-06 | 0.000268 | 3.51735 |
| EDEM3 | -1.65621 | 7.709573 | -3.99288 | 0.000126 | 0.002397 | 0.7929 |
| NOC3L | -1.65177 | 4.366532 | -5.64654 | 1.6E-07 | 0.0000154 | 7.092727 |
| SLC35F5 | -1.64378 | 7.146764 | -4.5207 | 0.0000173 | 0.000534 | 2.656221 |
| PRIMPOL | -1.63753 | 4.743439 | -6.5006 | 3.36E-09 | 6.51E-07 | 10.77892 |
| ORC2 | -1.6314 | 5.876704 | -6.83935 | 6.86E-10 | 0.0000002 | 12.29745 |
| ZNF721 | -1.62552 | 6.179571 | -4.615 | 0.0000119 | 0.000404 | 3.005165 |
| EXOC1 | -1.62427 | 6.439348 | -6.753 | 1.03E-09 | 2.61E-07 | 11.90778 |
| BNIP3 | -1.62311 | 5.297517 | -4.28153 | 0.0000433 | 0.001089 | 1.792466 |
| UFL1 | -1.62295 | 7.298496 | -5.02965 | 2.22E-06 | 0.000109 | 4.59175 |
| DENND5B | -1.61619 | 7.601779 | -5.84826 | 6.54E-08 | 7.54E-06 | 7.942269 |
| NEK7 | -1.6099 | 6.894926 | -4.70292 | 0.0000084 | 0.000309 | 3.334591 |
| ATG4C | -1.60628 | 5.877071 | -4.00795 | 0.000119 | 0.002298 | 0.843854 |
| NUP107 | -1.60249 | 6.218653 | -6.28424 | 9.11E-09 | 1.54E-06 | 9.82425 |
| ZDHHC13 | -1.59927 | 5.899307 | -5.21991 | 0.000001 | 0.0000595 | 5.346296 |
| ATP10D | -1.59387 | 6.612296 | -5.1063 | 1.61E-06 | 0.0000868 | 4.893812 |
| WDR89 | -1.59037 | 4.786059 | -5.67179 | 1.43E-07 | 0.0000143 | 7.19826 |
| SEC23B | -1.587 | 8.494306 | -5.2517 | 8.76E-07 | 0.0000533 | 5.473929 |
| BMPR1A | -1.58451 | 5.581458 | -4.27701 | 0.000044 | 0.001102 | 1.776449 |
| SOS1 | -1.58272 | 7.25341 | -4.69573 | 8.64E-06 | 0.000315 | 3.3075 |
| FBXO18 | -1.58236 | 7.390173 | -4.78435 | 6.06E-06 | 0.000241 | 3.643112 |
| NCAPD2 | -1.57699 | 6.279774 | -5.89957 | 5.2E-08 | 6.22E-06 | 8.160649 |
| ZNF260 | -1.57195 | 4.760984 | -5.7635 | 9.53E-08 | 0.0000104 | 7.583579 |
| TRAPPC11 | -1.56733 | 6.196359 | -6.25998 | 1.02E-08 | 1.65E-06 | 9.718035 |
| APPBP2 | -1.56432 | 6.626055 | -5.49187 | 3.13E-07 | 0.0000248 | 6.451345 |
| SMC6 | -1.56281 | 5.272997 | -5.22843 | 9.67E-07 | 0.0000577 | 5.380478 |
| TCEANC | -1.56059 | 4.814202 | -7.01067 | 3.04E-10 | 1.11E-07 | 13.07533 |
| ATRX | -1.55678 | 6.248 | -5.26195 | 8.39E-07 | 0.0000514 | 5.515141 |
| CDC6 | -1.5525 | 5.056455 | -4.83636 | 4.91E-06 | 0.000206 | 3.841875 |
| ZFP62 | -1.53625 | 5.142702 | -5.18132 | 1.18E-06 | 0.0000679 | 5.191969 |
| MSH2 | -1.52374 | 4.32844 | -4.92182 | 3.46E-06 | 0.000154 | 4.171256 |
| SEC24A | -1.51068 | 8.48999 | -6.0312 | 2.88E-08 | 3.85E-06 | 8.724743 |
| AKAP12 | -1.50915 | 5.566004 | -5.2845 | 7.62E-07 | 0.0000478 | 5.605997 |
| PBRM1 | -1.50856 | 7.293592 | -5.89927 | 5.21E-08 | 6.22E-06 | 8.159343 |
| SGK3 | -1.50838 | 6.45147 | -4.82623 | 5.11E-06 | 0.000211 | 3.803076 |
| HSD17B4 | -1.49625 | 6.755604 | -3.21255 | 0.00178 | 0.016356 | -1.64771 |
| DDX17 | -1.4955 | 9.194057 | -4.916 | 3.55E-06 | 0.000157 | 4.148727 |
| WDR11 | -1.49279 | 6.878563 | -5.48989 | 3.16E-07 | 0.0000249 | 6.443175 |
| KLHL5 | -1.49259 | 7.062374 | -4.49889 | 0.0000188 | 0.000571 | 2.576205 |
| COPB2 | -1.48909 | 8.092603 | -4.19222 | 0.0000606 | 0.001385 | 1.47804 |
| RSBN1L | -1.4878 | 6.321513 | -4.89299 | 0.0000039 | 0.00017 | 4.059743 |
| PDE7A | -1.48691 | 5.466515 | -5.51434 | 2.84E-07 | 0.000023 | 6.543964 |
| AKT3 | -1.48661 | 4.505485 | -4.0061 | 0.00012 | 0.00231 | 0.837608 |
| RFC1 | -1.48271 | 7.460724 | -6.1737 | 1.51E-08 | 2.28E-06 | 9.341564 |
| ACTR10 | -1.46885 | 6.672004 | -5.63491 | 1.68E-07 | 0.0000157 | 7.044199 |
| MAN1A1 | -1.46828 | 9.668447 | -4.63943 | 0.0000108 | 0.000375 | 3.096315 |
| FCHSD2 | -1.46571 | 8.125143 | -3.50026 | 0.000701 | 0.008343 | -0.79502 |
| OMA1 | -1.46455 | 6.826371 | -4.72791 | 0.0000076 | 0.000289 | 3.428916 |
| CDC16 | -1.46144 | 6.552794 | -4.65266 | 0.0000103 | 0.000362 | 3.145786 |
| ZC3H13 | -1.46075 | 6.274 | -6.51911 | 3.08E-09 | 6.13E-07 | 10.86116 |
| ZNF383 | -1.45433 | 5.541317 | -4.96387 | 2.91E-06 | 0.000134 | 4.334617 |
| AARS | -1.45427 | 7.914399 | -3.48047 | 0.000749 | 0.008722 | -0.85551 |
| RAB30 | -1.45325 | 8.599908 | -3.27601 | 0.001456 | 0.01431 | -1.46461 |
| TIAM1 | -1.45169 | 5.618413 | -4.12764 | 0.000077 | 0.001645 | 1.253507 |
| ACADM | -1.45113 | 7.71592 | -3.66705 | 0.000399 | 0.005646 | -0.27489 |
| GBF1 | -1.45104 | 7.589826 | -6.2363 | 1.13E-08 | 1.78E-06 | 9.614463 |
| CNOT1 | -1.44756 | 7.846792 | -5.02889 | 2.23E-06 | 0.000109 | 4.588763 |
| SUCLA2 | -1.44425 | 6.147656 | -5.15585 | 1.31E-06 | 0.0000736 | 5.090479 |
| BCL2L1 | -1.44419 | 7.732366 | -5.02052 | 2.31E-06 | 0.000112 | 4.555913 |
| HIBCH | -1.44417 | 4.949998 | -3.60172 | 0.000498 | 0.006599 | -0.4808 |
| ZCCHC11 | -1.43396 | 6.887547 | -6.26827 | 9.8E-09 | 1.63E-06 | 9.754312 |
| ATM | -1.43129 | 7.308799 | -4.24031 | 0.0000506 | 0.00122 | 1.646788 |
| ATF2 | -1.4265 | 8.117698 | -6.982 | 3.49E-10 | 1.21E-07 | 12.94473 |
| USP28 | -1.42466 | 5.798352 | -5.10227 | 1.64E-06 | 0.0000879 | 4.877876 |
| PIP4K2A | -1.42258 | 7.585344 | -4.09231 | 0.0000877 | 0.001832 | 1.131711 |
| SESN3 | -1.42235 | 5.594122 | -3.24327 | 0.001616 | 0.015265 | -1.55943 |
| TTC3 | -1.42217 | 7.295136 | -5.43129 | 4.07E-07 | 0.0000299 | 6.202592 |
| CEP97 | -1.41866 | 6.758066 | -4.11674 | 0.0000802 | 0.001701 | 1.215855 |
| PRRC2A | -1.41224 | 7.336008 | -5.19313 | 1.12E-06 | 0.0000654 | 5.239148 |
| ABCD3 | -1.40721 | 6.032327 | -4.39356 | 0.0000282 | 0.00078 | 2.193185 |
| SLC44A1 | -1.40678 | 8.034155 | -4.04978 | 0.000103 | 0.002063 | 0.986064 |
| KIAA0319L | -1.40498 | 6.862807 | -6.29172 | 8.8E-09 | 0.0000015 | 9.857041 |
| STX7 | -1.40493 | 6.794469 | -4.29931 | 0.0000405 | 0.00103 | 1.855601 |
| TMEM131 | -1.40428 | 7.263591 | -5.29214 | 7.38E-07 | 0.0000465 | 5.636862 |
| AGL | -1.40381 | 5.274448 | -4.64941 | 0.0000104 | 0.000364 | 3.133607 |
| LARP1B | -1.40346 | 6.38503 | -5.21882 | 1.01E-06 | 0.0000596 | 5.341948 |
| BMP2K | -1.39496 | 5.983931 | -5.28153 | 7.72E-07 | 0.0000482 | 5.594026 |
| GOLGA5 | -1.39265 | 6.720102 | -5.16824 | 1.25E-06 | 0.0000705 | 5.139826 |
| GFPT1 | -1.3909 | 8.860628 | -4.89423 | 3.88E-06 | 0.00017 | 4.064546 |
| C2orf42 | -1.38596 | 5.781022 | -6.58631 | 2.25E-09 | 5.04E-07 | 11.1605 |
| INPP4A | -1.37986 | 7.870158 | -4.73781 | 0.0000073 | 0.00028 | 3.466387 |
| SMARCAD1 | -1.3793 | 5.478758 | -4.65013 | 0.0000104 | 0.000363 | 3.136297 |
| ZNF566 | -1.37893 | 4.037014 | -6.13887 | 1.77E-08 | 2.59E-06 | 9.19023 |
| IBTK | -1.37285 | 6.813958 | -4.70872 | 0.0000082 | 0.000303 | 3.356436 |
| MIS12 | -1.37213 | 6.37579 | -5.4773 | 3.33E-07 | 0.0000257 | 6.391367 |
| ASH2L | -1.36658 | 7.283635 | -5.38434 | 4.98E-07 | 0.000035 | 6.010828 |
| ZMYM4 | -1.36599 | 6.474478 | -6.57128 | 2.41E-09 | 5.33E-07 | 11.09344 |
| SEC31A | -1.36127 | 9.168677 | -5.34542 | 5.88E-07 | 0.0000397 | 5.852561 |
| CEPT1 | -1.35587 | 7.04221 | -4.18341 | 0.0000626 | 0.001419 | 1.447274 |
| FBXL4 | -1.35399 | 5.655859 | -5.33299 | 6.2E-07 | 0.0000412 | 5.802123 |
| DNAJC3 | -1.35089 | 9.068645 | -5.1992 | 1.09E-06 | 0.0000641 | 5.263418 |
| GCC2 | -1.34881 | 7.434423 | -5.12427 | 0.0000015 | 0.0000824 | 4.965028 |
| IPO8 | -1.34849 | 6.682736 | -4.66031 | 9.95E-06 | 0.000352 | 3.174424 |
| BLZF1 | -1.34418 | 6.72857 | -4.50129 | 0.0000186 | 0.000567 | 2.584993 |
| MGA | -1.34287 | 5.259348 | -6.13415 | 1.81E-08 | 2.61E-06 | 9.169729 |
| ERCC5 | -1.34073 | 6.811672 | -5.58294 | 2.11E-07 | 0.0000184 | 6.827905 |
| CCDC117 | -1.33975 | 7.941991 | -6.01598 | 3.09E-08 | 4.08E-06 | 8.659226 |
| RNASEL | -1.33952 | 4.430252 | -5.95652 | 4.03E-08 | 4.99E-06 | 8.40398 |
| ERLEC1 | -1.33699 | 9.187089 | -4.18674 | 0.0000618 | 0.001404 | 1.458891 |
| CHD9 | -1.33194 | 6.641041 | -5.30822 | 6.89E-07 | 0.0000443 | 5.701823 |
| LIMD1 | -1.33171 | 6.889407 | -5.09164 | 1.72E-06 | 0.000091 | 4.835834 |
| MEGF9 | -1.33104 | 7.203514 | -3.16502 | 0.002066 | 0.01827 | -1.78294 |
| HEATR5B | -1.3297 | 6.297355 | -5.24482 | 9.02E-07 | 0.0000547 | 5.446249 |
| IDH1 | -1.32964 | 6.454925 | -3.51988 | 0.000656 | 0.007948 | -0.7348 |
| HDAC2 | -1.32833 | 6.945289 | -5.16517 | 1.26E-06 | 0.000071 | 5.127608 |
| ARMCX3 | -1.32541 | 6.998762 | -5.26353 | 8.33E-07 | 0.0000512 | 5.521521 |
| CHAMP1 | -1.32189 | 5.652016 | -4.28984 | 0.0000419 | 0.00106 | 1.821942 |
| XPO7 | -1.31804 | 6.736176 | -5.10575 | 1.62E-06 | 0.0000868 | 4.891659 |
| HSDL2 | -1.31764 | 6.259533 | -3.4994 | 0.000703 | 0.008354 | -0.79766 |
| CYP1B1 | -1.31682 | 5.569789 | -2.81423 | 0.005909 | 0.037627 | -2.72952 |
| TOP2B | -1.31585 | 7.167519 | -4.41281 | 0.0000262 | 0.000735 | 2.262735 |
| MTIF3 | -1.31286 | 6.51324 | -5.14952 | 1.35E-06 | 0.0000752 | 5.065297 |
| UBR2 | -1.31193 | 7.53532 | -5.13032 | 1.46E-06 | 0.0000808 | 4.989032 |
| GRAMD1C | -1.31133 | 5.617732 | -3.39695 | 0.000986 | 0.010772 | -1.10782 |
| GOPC | -1.30834 | 6.125229 | -4.98856 | 2.63E-06 | 0.000125 | 4.430878 |
| ZC3H6 | -1.30758 | 6.349985 | -5.09867 | 1.67E-06 | 0.0000888 | 4.863651 |
| CYFIP2 | -1.30457 | 6.972977 | -3.74357 | 0.000306 | 0.00465 | -0.03012 |
| SLC33A1 | -1.30385 | 8.142989 | -4.32735 | 0.0000364 | 0.000949 | 1.955544 |
| MYCBP2 | -1.30087 | 6.899678 | -4.34982 | 0.0000334 | 0.000892 | 2.035912 |
| ALG11 | -1.29534 | 6.560906 | -3.997 | 0.000124 | 0.002371 | 0.80681 |
| RASA1 | -1.29254 | 6.901647 | -4.33938 | 0.0000347 | 0.000921 | 1.998523 |
| C10orf88 | -1.29125 | 4.320426 | -6.77921 | 9.11E-10 | 2.47E-07 | 12.0259 |
| ZNF652 | -1.29085 | 6.764886 | -6.96402 | 3.8E-10 | 1.3E-07 | 12.86289 |
| RAD50 | -1.28673 | 6.574914 | -4.20224 | 0.0000583 | 0.001346 | 1.513101 |
| ANKRD46 | -1.28401 | 5.603715 | -4.1583 | 0.0000687 | 0.001514 | 1.359816 |
| FUT8 | -1.28323 | 8.495533 | -2.72857 | 0.007539 | 0.044189 | -2.94645 |
| RSPRY1 | -1.28133 | 6.810443 | -5.06466 | 1.92E-06 | 0.0000983 | 4.72937 |
| ETFDH | -1.27812 | 5.816471 | -4.83571 | 4.92E-06 | 0.000206 | 3.839366 |
| PREB | -1.27494 | 7.69579 | -4.78237 | 0.0000061 | 0.000242 | 3.635578 |
| GPR180 | -1.27303 | 5.518363 | -4.1875 | 0.0000616 | 0.001402 | 1.461554 |
| ZZZ3 | -1.27259 | 6.533227 | -5.81273 | 7.66E-08 | 8.62E-06 | 7.791605 |
| BBS10 | -1.27252 | 4.277245 | -6.25919 | 1.02E-08 | 1.65E-06 | 9.714541 |
| CPSF2 | -1.27128 | 7.414622 | -4.25109 | 0.0000486 | 0.001181 | 1.684808 |
| CKAP5 | -1.26399 | 6.291687 | -4.70119 | 8.46E-06 | 0.00031 | 3.32804 |
| SLC30A5 | -1.26397 | 6.693791 | -4.35096 | 0.0000332 | 0.000891 | 2.039997 |
| DDIAS | -1.26026 | 4.066857 | -7.96117 | 3.06E-12 | 2.45E-09 | 17.48445 |
| KRIT1 | -1.25941 | 6.568349 | -5.68903 | 1.32E-07 | 0.0000136 | 7.270459 |
| SF3B3 | -1.25731 | 7.450967 | -5.46817 | 3.47E-07 | 0.0000267 | 6.353851 |
| TRAPPC6B | -1.25637 | 6.041755 | -4.26541 | 0.000046 | 0.001138 | 1.735384 |
| UBE4B | -1.25583 | 7.201471 | -6.16222 | 1.59E-08 | 2.37E-06 | 9.291666 |
| RTCA | -1.25515 | 7.489211 | -5.07283 | 1.86E-06 | 0.0000963 | 4.761596 |
| DEK | -1.25482 | 7.226603 | -2.82705 | 0.005695 | 0.036666 | -2.69657 |
| EIF5B | -1.25036 | 7.735285 | -4.92683 | 3.39E-06 | 0.000152 | 4.190697 |
| ZNF146 | -1.24777 | 6.305315 | -3.40285 | 0.000967 | 0.010621 | -1.09015 |
| KIAA1551 | -1.24641 | 8.051699 | -3.83689 | 0.000221 | 0.003613 | 0.273386 |
| SLC27A2 | -1.24546 | 4.318821 | -3.24154 | 0.001625 | 0.015297 | -1.56442 |
| STK38 | -1.24455 | 6.602467 | -3.60153 | 0.000499 | 0.006599 | -0.48141 |
| C9orf64 | -1.24423 | 5.957547 | -5.17328 | 1.22E-06 | 0.0000696 | 5.159922 |
| ATXN2 | -1.24305 | 6.830114 | -7.45755 | 3.56E-11 | 2.01E-08 | 15.1309 |
| ARFIP1 | -1.2422 | 6.292162 | -4.09689 | 0.0000863 | 0.001807 | 1.147481 |
| SMC1A | -1.24122 | 6.402789 | -5.0738 | 1.85E-06 | 0.0000961 | 4.765396 |
| EFCAB14 | -1.24024 | 7.874242 | -6.09374 | 2.17E-08 | 2.99E-06 | 8.994697 |
| LTN1 | -1.23858 | 6.368817 | -4.45034 | 0.0000227 | 0.000661 | 2.398914 |
| HMBOX1 | -1.23831 | 5.779605 | -5.98917 | 3.48E-08 | 4.48E-06 | 8.544002 |
| ATG2B | -1.23665 | 5.969583 | -5.02381 | 2.28E-06 | 0.000111 | 4.56882 |
| HECTD1 | -1.23562 | 7.206763 | -5.55257 | 2.41E-07 | 0.0000205 | 6.70197 |
| ESF1 | -1.23204 | 5.231943 | -4.84373 | 4.76E-06 | 0.000201 | 3.870147 |
| PTER | -1.23108 | 6.444012 | -5.97468 | 3.72E-08 | 0.0000047 | 8.481818 |
| PIBF1 | -1.23008 | 4.078869 | -4.78769 | 5.97E-06 | 0.000239 | 3.65583 |
| DPY19L4 | -1.22991 | 5.127744 | -4.87061 | 4.27E-06 | 0.000185 | 3.973461 |
| NFX1 | -1.22832 | 7.781456 | -4.92937 | 3.36E-06 | 0.000151 | 4.200516 |
| ZNF140 | -1.22496 | 4.106332 | -6.64345 | 1.72E-09 | 4.09E-07 | 11.41592 |
| BORA | -1.22195 | 3.923547 | -6.29084 | 8.84E-09 | 0.0000015 | 9.853173 |
| RABEP1 | -1.21994 | 6.982328 | -6.12011 | 1.93E-08 | 2.75E-06 | 9.108866 |
| ZNF302 | -1.21849 | 6.065225 | -4.05488 | 0.000101 | 0.002034 | 1.003466 |
| EPB41 | -1.21596 | 7.563881 | -5.58047 | 2.13E-07 | 0.0000185 | 6.81765 |
| RALGAPB | -1.21384 | 7.294934 | -6.32459 | 7.57E-09 | 1.31E-06 | 10.0013 |
| IDE | -1.21234 | 7.450409 | -4.90963 | 3.64E-06 | 0.000161 | 4.124074 |
| MLEC | -1.21206 | 7.849926 | -3.94143 | 0.000152 | 0.002737 | 0.619908 |
| ACAT1 | -1.21049 | 7.004264 | -4.18586 | 0.000062 | 0.001407 | 1.455829 |
| NEMF | -1.20918 | 6.504294 | -5.3648 | 5.41E-07 | 0.0000376 | 5.931275 |
| ZNF780B | -1.20887 | 5.946806 | -3.39405 | 0.000995 | 0.010834 | -1.11648 |
| HOOK3 | -1.20817 | 7.132884 | -4.78133 | 6.13E-06 | 0.000242 | 3.631628 |
| TRAM2 | -1.20635 | 8.100282 | -3.96625 | 0.000139 | 0.002579 | 0.703167 |
| DCP1B | -1.20578 | 4.251687 | -9.10272 | 1.06E-14 | 2.08E-11 | 22.90667 |
| ZNF420 | -1.20486 | 4.259006 | -4.92551 | 3.41E-06 | 0.000153 | 4.185581 |
| ELP3 | -1.20465 | 6.551736 | -4.86216 | 4.42E-06 | 0.00019 | 3.940952 |
| BPTF | -1.20224 | 6.869145 | -5.61459 | 1.84E-07 | 0.0000168 | 6.959507 |
| CHST15 | -1.1992 | 7.598644 | -6.22831 | 1.18E-08 | 1.83E-06 | 9.579604 |
| AHCYL1 | -1.19507 | 6.680754 | -5.43989 | 3.92E-07 | 0.000029 | 6.237832 |
| ANAPC7 | -1.19376 | 6.112411 | -5.3626 | 5.46E-07 | 0.0000378 | 5.922349 |
| ALDH18A1 | -1.19259 | 7.798896 | -3.46489 | 0.000788 | 0.009063 | -0.90293 |
| GOLGA4 | -1.19071 | 7.262936 | -4.30395 | 0.0000398 | 0.001015 | 1.872105 |
| TRIO | -1.18667 | 5.994327 | -3.89992 | 0.000176 | 0.003029 | 0.481515 |
| SEC14L1 | -1.18663 | 8.290792 | -5.54445 | 2.49E-07 | 0.0000209 | 6.668353 |
| APAF1 | -1.18525 | 5.911834 | -3.31031 | 0.001305 | 0.013271 | -1.36445 |
| B4GALT1 | -1.18355 | 8.819268 | -3.29663 | 0.001363 | 0.013651 | -1.4045 |
| IPP | -1.18189 | 5.293839 | -5.05642 | 1.99E-06 | 0.000101 | 4.696953 |
| TAOK1 | -1.18061 | 7.266964 | -4.67525 | 9.38E-06 | 0.000337 | 3.230468 |
| ATP6V1C1 | -1.1794 | 6.208149 | -3.25118 | 0.001576 | 0.015081 | -1.53657 |
| AP5M1 | -1.17796 | 6.712885 | -4.39451 | 0.0000281 | 0.000778 | 2.196638 |
| CTR9 | -1.17752 | 7.644728 | -4.45233 | 0.0000225 | 0.000657 | 2.406169 |
| ZNF493 | -1.17651 | 3.751186 | -3.92222 | 0.000163 | 0.002864 | 0.555718 |
| IGFBP5 | -1.17381 | 4.927926 | -3.24153 | 0.001625 | 0.015297 | -1.56445 |
| PAPOLG | -1.1738 | 6.503582 | -4.65197 | 0.0000103 | 0.000362 | 3.143217 |
| ZNF570 | -1.17098 | 4.108518 | -4.72572 | 7.67E-06 | 0.000291 | 3.420642 |
| PHKB | -1.17035 | 7.17212 | -4.04607 | 0.000104 | 0.002083 | 0.973408 |
| TBCK | -1.16938 | 5.695893 | -3.85629 | 0.000206 | 0.003441 | 0.337191 |
| FLNB | -1.16826 | 6.042727 | -4.69596 | 8.63E-06 | 0.000315 | 3.30834 |
| RAB5B | -1.1682 | 7.120475 | -5.56412 | 2.29E-07 | 0.0000198 | 6.749819 |
| ZDBF2 | -1.1665 | 5.359155 | -2.97878 | 0.003647 | 0.027158 | -2.29698 |
| GPR75-ASB3 | -1.16607 | 7.751153 | -3.70858 | 0.000345 | 0.005094 | -0.1425 |
| TIMP2 | -1.16508 | 8.14806 | -2.88887 | 0.004758 | 0.032492 | -2.53586 |
| C5orf42 | -1.16486 | 4.536705 | -6.91237 | 4.86E-10 | 1.55E-07 | 12.62825 |
| ATP7A | -1.16286 | 5.528893 | -5.62951 | 1.72E-07 | 0.000016 | 7.021645 |
| NUCB2 | -1.1603 | 10.17214 | -4.00832 | 0.000119 | 0.002298 | 0.845117 |
| PIK3R4 | -1.15962 | 6.026298 | -5.19985 | 1.09E-06 | 0.0000641 | 5.265992 |
| DCAF7 | -1.15651 | 6.693518 | -5.99965 | 3.32E-08 | 4.34E-06 | 8.588997 |
| TMEM56 | -1.15412 | 7.19807 | -3.27455 | 0.001463 | 0.014358 | -1.46885 |
| PARN | -1.15386 | 7.413489 | -5.61363 | 1.84E-07 | 0.0000168 | 6.955502 |
| TMEM106B | -1.15372 | 7.173674 | -3.56092 | 0.000572 | 0.007238 | -0.60799 |
| EPS15 | -1.15355 | 8.782533 | -5.79146 | 8.42E-08 | 9.38E-06 | 7.701641 |
| METTL4 | -1.15076 | 5.872139 | -3.95847 | 0.000143 | 0.002633 | 0.677032 |
| SEL1L | -1.15073 | 10.19015 | -3.05646 | 0.002886 | 0.02303 | -2.08566 |
| CBX5 | -1.14819 | 6.919918 | -3.48445 | 0.000739 | 0.008651 | -0.84334 |
| ZMYM1 | -1.14778 | 4.009175 | -6.77348 | 9.36E-10 | 2.51E-07 | 12.00004 |
| PUS7L | -1.14593 | 6.10828 | -3.79086 | 0.000259 | 0.004102 | 0.123 |
| NXPE3 | -1.14495 | 7.894391 | -3.69772 | 0.000359 | 0.005229 | -0.17722 |
| ARHGAP12 | -1.13849 | 6.850082 | -4.51343 | 0.0000177 | 0.000544 | 2.62953 |
| TERF1 | -1.13758 | 7.008336 | -4.04669 | 0.000104 | 0.002081 | 0.975531 |
| VTA1 | -1.13562 | 6.146306 | -4.47762 | 0.0000204 | 0.000608 | 2.498367 |
| PSIP1 | -1.13557 | 5.651334 | -3.52853 | 0.000638 | 0.007806 | -0.70815 |
| USP33 | -1.13435 | 6.759632 | -4.76253 | 6.61E-06 | 0.000259 | 3.5601 |
| MRPS31 | -1.13256 | 5.906088 | -4.56537 | 0.0000145 | 0.000473 | 2.820924 |
| SEC24C | -1.13086 | 7.023777 | -6.33133 | 7.34E-09 | 1.29E-06 | 10.03093 |
| CAB39 | -1.13073 | 8.975419 | -4.17042 | 0.0000657 | 0.001467 | 1.401995 |
| BTRC | -1.12927 | 5.466211 | -6.47567 | 3.77E-09 | 7.19E-07 | 10.66825 |
| S100PBP | -1.12693 | 6.500339 | -4.83551 | 4.92E-06 | 0.000206 | 3.838626 |
| NUP37 | -1.1262 | 5.685269 | -4.34772 | 0.0000337 | 0.000898 | 2.028407 |
| RPF2 | -1.12584 | 6.369589 | -3.89677 | 0.000178 | 0.003052 | 0.47105 |
| GLDC | -1.12441 | 6.138372 | -2.854 | 0.005268 | 0.034859 | -2.62687 |
| DROSHA | -1.12247 | 5.562063 | -3.90371 | 0.000174 | 0.002999 | 0.494109 |
| CREB1 | -1.11897 | 7.871826 | -5.32313 | 6.47E-07 | 0.0000423 | 5.762154 |
| SLC26A2 | -1.11834 | 5.197084 | -4.59447 | 0.0000129 | 0.000431 | 2.928783 |
| PAK2 | -1.116 | 7.305336 | -3.68775 | 0.000371 | 0.005372 | -0.20904 |
| ERLIN1 | -1.11473 | 6.894591 | -3.16711 | 0.002052 | 0.018203 | -1.77704 |
| SYVN1 | -1.10988 | 8.436698 | -4.7032 | 8.39E-06 | 0.000309 | 3.335647 |
| SRRM2 | -1.10712 | 8.268747 | -3.99618 | 0.000125 | 0.002375 | 0.804041 |
| SPG11 | -1.10405 | 6.508771 | -4.13485 | 0.000075 | 0.001612 | 1.278465 |
| LBR | -1.10379 | 8.355467 | -3.20973 | 0.001796 | 0.016469 | -1.65575 |
| FGD4 | -1.10247 | 5.945941 | -3.26231 | 0.001521 | 0.014791 | -1.50437 |
| ZMYND8 | -1.10207 | 6.469452 | -5.38651 | 4.93E-07 | 0.000035 | 6.019675 |
| MIS18BP1 | -1.09925 | 5.584293 | -3.81417 | 0.000239 | 0.003861 | 0.198998 |
| UFSP2 | -1.09678 | 5.626495 | -3.24264 | 0.001619 | 0.015278 | -1.56125 |
| HEATR5A | -1.09508 | 5.05095 | -5.06835 | 1.89E-06 | 0.0000974 | 4.743916 |
| CHM | -1.09409 | 4.425857 | -5.38731 | 4.91E-07 | 0.000035 | 6.022958 |
| WDR36 | -1.09301 | 6.703158 | -4.39922 | 0.0000276 | 0.00077 | 2.213626 |
| NUP43 | -1.09263 | 6.215445 | -3.04343 | 0.003002 | 0.023636 | -2.12144 |
| TBP | -1.09099 | 6.50083 | -5.74028 | 1.06E-07 | 0.0000113 | 7.485728 |
| ZNF117 | -1.09016 | 5.323342 | -4.71212 | 8.09E-06 | 0.000301 | 3.369258 |
| DICER1 | -1.08975 | 6.999479 | -4.1641 | 0.0000673 | 0.001493 | 1.379976 |
| BTN3A2 | -1.08869 | 7.979155 | -3.11269 | 0.002429 | 0.020364 | -1.92995 |
| ZNF275 | -1.0884 | 7.712779 | -3.14093 | 0.002226 | 0.019226 | -1.85086 |
| PRIM1 | -1.08808 | 5.799173 | -4.05887 | 0.0000992 | 0.00201 | 1.01712 |
| FADS1 | -1.0857 | 4.938832 | -4.5113 | 0.0000179 | 0.000548 | 2.621704 |
| IWS1 | -1.08498 | 6.005749 | -6.60773 | 2.04E-09 | 4.74E-07 | 11.25616 |
| GTF2I | -1.08455 | 7.535607 | -3.81856 | 0.000235 | 0.003815 | 0.213343 |
| CLCC1 | -1.084 | 7.997054 | -3.62015 | 0.000468 | 0.006305 | -0.423 |
| TBC1D9 | -1.08372 | 9.083823 | -3.05447 | 0.002903 | 0.023083 | -2.09113 |
| EHHADH | -1.0835 | 4.427784 | -5.15332 | 1.33E-06 | 0.0000742 | 5.080424 |
| FRYL | -1.07604 | 5.782458 | -4.5482 | 0.0000155 | 0.000494 | 2.75751 |
| HERC4 | -1.07539 | 5.785855 | -3.71638 | 0.000336 | 0.004986 | -0.11754 |
| USP6NL | -1.07399 | 6.758741 | -2.94024 | 0.004091 | 0.02932 | -2.40013 |
| THOC2 | -1.07335 | 6.795578 | -3.50547 | 0.000689 | 0.008237 | -0.77904 |
| AAAS | -1.07321 | 6.649968 | -5.46517 | 3.51E-07 | 0.0000269 | 6.34153 |
| ZNF518A | -1.07235 | 5.486204 | -3.6521 | 0.00042 | 0.005878 | -0.32225 |
| CDC23 | -1.07203 | 6.255574 | -4.47059 | 0.000021 | 0.000621 | 2.472689 |
| SETD5 | -1.07159 | 7.714158 | -5.03385 | 2.18E-06 | 0.000108 | 4.608215 |
| EHBP1 | -1.07145 | 5.545075 | -4.96876 | 2.86E-06 | 0.000133 | 4.353645 |
| NSUN6 | -1.071 | 4.806657 | -4.26423 | 0.0000462 | 0.00114 | 1.73122 |
| AP3M1 | -1.06986 | 7.124677 | -5.38515 | 4.96E-07 | 0.000035 | 6.014151 |
| XPO1 | -1.06889 | 7.323256 | -3.12054 | 0.002371 | 0.020082 | -1.90803 |
| TUBD1 | -1.06726 | 4.755682 | -5.48326 | 3.25E-07 | 0.0000253 | 6.415891 |
| MAPK9 | -1.06668 | 7.65276 | -4.62754 | 0.0000113 | 0.000387 | 3.051901 |
| ENTPD4 | -1.06592 | 8.088887 | -4.63896 | 0.0000108 | 0.000375 | 3.094536 |
| TP53 | -1.06523 | 6.411874 | -4.5638 | 0.0000146 | 0.000475 | 2.815143 |
| RGL1 | -1.06406 | 5.562217 | -3.16382 | 0.002074 | 0.01831 | -1.78634 |
| ZBTB38 | -1.06316 | 10.14994 | -3.33544 | 0.001203 | 0.012474 | -1.29056 |
| 44993 | -1.06044 | 7.824591 | -4.33239 | 0.0000357 | 0.000937 | 1.973546 |
| TTC37 | -1.05992 | 7.12223 | -2.87994 | 0.004884 | 0.033064 | -2.55926 |
| NCOA3 | -1.05983 | 9.353332 | -3.24861 | 0.001589 | 0.01512 | -1.544 |
| DARS2 | -1.05838 | 6.183081 | -3.24885 | 0.001587 | 0.01512 | -1.5433 |
| RBBP4 | -1.05829 | 9.204423 | -4.19094 | 0.0000608 | 0.00139 | 1.473558 |
| CTC1 | -1.0565 | 6.651629 | -7.75641 | 8.33E-12 | 6.2E-09 | 16.52356 |
| KDM4A | -1.0524 | 6.349991 | -5.88176 | 5.63E-08 | 6.63E-06 | 8.084728 |
| PIKFYVE | -1.05237 | 6.549893 | -5.03571 | 2.17E-06 | 0.000108 | 4.615527 |
| ZNF680 | -1.05208 | 3.477699 | -5.59984 | 1.96E-07 | 0.0000175 | 6.898121 |
| POLH | -1.05178 | 5.84937 | -5.16799 | 1.25E-06 | 0.0000705 | 5.138828 |
| ZDHHC6 | -1.05094 | 6.875638 | -4.22634 | 0.0000533 | 0.001267 | 1.597631 |
| FAM126A | -1.05088 | 6.351534 | -2.80906 | 0.005997 | 0.037882 | -2.74279 |
| MYO5A | -1.0503 | 7.407296 | -3.56751 | 0.000559 | 0.007126 | -0.58751 |
| ZBTB40 | -1.04984 | 5.682094 | -4.98389 | 2.68E-06 | 0.000127 | 4.412641 |
| STAM2 | -1.04965 | 6.124043 | -4.73795 | 0.0000073 | 0.00028 | 3.466921 |
| DHX36 | -1.04767 | 6.3069 | -3.55779 | 0.000578 | 0.00729 | -0.6177 |
| TOP1 | -1.04634 | 8.682019 | -4.06356 | 0.0000975 | 0.001984 | 1.03316 |
| ZNF254 | -1.0453 | 5.308969 | -4.01765 | 0.000115 | 0.00224 | 0.876757 |
| RNFT1 | -1.04495 | 7.327508 | -3.4195 | 0.000915 | 0.010208 | -1.04015 |
| SACM1L | -1.04352 | 7.606214 | -3.12883 | 0.002311 | 0.019726 | -1.88483 |
| NAT10 | -1.04315 | 5.980166 | -4.46796 | 0.0000212 | 0.000625 | 2.463105 |
| INTS2 | -1.04188 | 5.856003 | -4.53869 | 0.0000161 | 0.000507 | 2.722421 |
| LARP4 | -1.04063 | 7.348013 | -4.27685 | 0.0000441 | 0.001102 | 1.77588 |
| IPO11 | -1.03942 | 6.513019 | -3.57917 | 0.000538 | 0.00696 | -0.55124 |
| PGM3 | -1.03876 | 7.70994 | -2.83528 | 0.005561 | 0.036104 | -2.67534 |
| TOP2A | -1.03651 | 5.263928 | -2.85943 | 0.005185 | 0.0345 | -2.61277 |
| ZMYM2 | -1.03529 | 6.740534 | -4.10551 | 0.0000836 | 0.001759 | 1.17716 |
| NSL1 | -1.03497 | 6.907617 | -3.41557 | 0.000927 | 0.010315 | -1.05197 |
| PIK3C2A | -1.03471 | 6.982785 | -3.74043 | 0.000309 | 0.004679 | -0.04026 |
| VPS35 | -1.03409 | 7.352052 | -4.08388 | 0.0000905 | 0.001878 | 1.102767 |
| BICD1 | -1.03223 | 6.680242 | -4.96549 | 2.89E-06 | 0.000134 | 4.340902 |
| ZNF616 | -1.0308 | 3.585915 | -7.05553 | 2.46E-10 | 9.23E-08 | 13.28002 |
| TMEM41B | -1.03074 | 6.27467 | -3.83842 | 0.000219 | 0.003605 | 0.278418 |
| RIF1 | -1.02958 | 7.031732 | -4.25943 | 0.0000471 | 0.001152 | 1.714251 |
| TMEM165 | -1.02945 | 7.955496 | -4.03061 | 0.00011 | 0.002164 | 0.920775 |
| GTF2H1 | -1.02908 | 5.60521 | -3.93175 | 0.000157 | 0.002798 | 0.587531 |
| GOLGA3 | -1.02769 | 6.91916 | -7.31109 | 7.22E-11 | 3.23E-08 | 14.45334 |
| NOL11 | -1.02661 | 5.094476 | -5.16728 | 1.25E-06 | 0.0000705 | 5.135995 |
| ZNF217 | -1.0262 | 7.828992 | -3.33162 | 0.001218 | 0.012589 | -1.30182 |
| SCARB2 | -1.02613 | 8.279451 | -3.6455 | 0.000429 | 0.005948 | -0.3431 |
| ABCC1 | -1.02387 | 6.739005 | -5.01267 | 2.38E-06 | 0.000116 | 4.525169 |
| RNF219 | -1.02348 | 5.276547 | -3.63644 | 0.000443 | 0.006073 | -0.3717 |
| BRCC3 | -1.02168 | 5.537461 | -4.33798 | 0.0000349 | 0.000925 | 1.993527 |
| STAT5B | -1.02126 | 5.930504 | -3.58215 | 0.000532 | 0.006913 | -0.54194 |
| ASCC3 | -1.02122 | 6.395879 | -3.29792 | 0.001358 | 0.013618 | -1.40074 |
| AQR | -1.02111 | 6.841108 | -4.11105 | 0.0000819 | 0.001731 | 1.196228 |
| RPAP3 | -1.0211 | 4.766763 | -3.9338 | 0.000156 | 0.002788 | 0.594367 |
| GEMIN5 | -1.02101 | 5.170115 | -4.99993 | 2.51E-06 | 0.000121 | 4.475304 |
| ATP6V0A1 | -1.01984 | 7.750672 | -3.94052 | 0.000152 | 0.002741 | 0.616858 |
| KPNB1 | -1.01895 | 7.844699 | -5.64196 | 1.63E-07 | 0.0000155 | 7.073613 |
| CLUAP1 | -1.01568 | 4.87507 | -7.18469 | 1.33E-10 | 5.61E-08 | 13.87154 |
| OPN3 | -1.01363 | 6.280029 | -4.74817 | 0.000007 | 0.00027 | 3.505639 |
| CASP8AP2 | -1.01171 | 5.36334 | -4.55834 | 0.0000149 | 0.00048 | 2.79496 |
| NT5DC2 | -1.01121 | 7.142408 | -2.7407 | 0.007286 | 0.043172 | -2.9161 |
| FBXO16 | -1.0107 | 4.863799 | -3.0639 | 0.002821 | 0.022654 | -2.0652 |
| ARSA | -1.00943 | 6.845355 | -4.13309 | 0.0000755 | 0.001618 | 1.272373 |
| KNTC1 | -1.00833 | 6.552593 | -2.81385 | 0.005915 | 0.037657 | -2.73049 |
| GCLC | -1.00807 | 6.926659 | -3.94904 | 0.000148 | 0.002693 | 0.645375 |
| KTN1 | -1.00746 | 6.918113 | -3.96765 | 0.000138 | 0.002572 | 0.707852 |
| VPS41 | -1.00744 | 7.059847 | -3.05214 | 0.002924 | 0.023224 | -2.09756 |
| TFCP2 | -1.00717 | 6.234638 | -3.89042 | 0.000182 | 0.003115 | 0.449973 |
| TIMMDC1 | -1.00647 | 7.419938 | -2.79939 | 0.006166 | 0.038567 | -2.7675 |
| CUL4A | -1.00627 | 6.270811 | -4.26883 | 0.0000454 | 0.001126 | 1.747478 |
| KITLG | -1.00567 | 3.040365 | -4.9247 | 3.42E-06 | 0.000153 | 4.182441 |
| SLC44A2 | -1.00382 | 7.178875 | -2.94886 | 0.003987 | 0.028793 | -2.37716 |
| ZKSCAN1 | -1.00343 | 6.186153 | -4.7119 | 0.0000081 | 0.000301 | 3.368429 |
| TMA16 | -1.00204 | 5.858446 | -4.11009 | 0.0000822 | 0.001734 | 1.19292 |
| MED23 | -1.00198 | 6.550309 | -3.83428 | 0.000223 | 0.003642 | 0.264846 |
| UTP20 | -1.00126 | 5.357981 | -4.4784 | 0.0000203 | 0.000607 | 2.501223 |
| LYST | -0.10009 | 5.1419064 | -2.915942 | 0.0054232 | 0.0334085 | -2.628587 |

**Supplementary Table 3. Enrichment analysis of the overlapped genes**

| **Term** | **Description** | **LogP** | **Symbols** |
| --- | --- | --- | --- |
| R-HSA-1640170 | Cell Cycle | -14.6153 | ATM,ATRX,CDC6,KPNB1,LBR,ORC2,PRIM1,RB1,RBBP4,RFC1,TERF1,TOP2A,TP53,XPO1,AAAS,SMC1A,BLZF1,USO1,CDC23,CDC16,BTRC,KNTC1,CKAP5,NCAPD2,AKT3,RAD50,ORC3,NSL1,ANAPC7,MIS18BP1,NUP107,TAOK1,MIS12,NUP37,BRCC3,BORA,CTC1,CABLES1,NEK7,NUP43,EPB41,RASA1,TOP1,ROCK2,EXOC1,SETDB2,ATF2,NEMF,IPO8,AHCYL1,XPO7,IPO11,IWS1,THOC2,SPG11,HDAC2,TOP2B,CASP8AP2,DDX17,CBX5,SMC6,BICD1,ATXN2,GTF2A1,GTF2H1,PAK2,TBP,PSIP1,VTA1,KTN1,CYFIP2,PIK3R4,GOPC,FLNB,RNASEL,FUT8,SEC24C,SEC24A,GEMIN5,VPS41,SLC33A1,CPSF2,SLC26A2,SEL1L,ERLIN1,ERLEC1,DICER1,DROSHA,DNAJC3,AQR,SF3B3,SRRM2,AGL,VCAN,B4GALT1,PHKB,CHST15,APAF1,BCL2L1,ATG2B,ACTR10,CREB1 |
| GO:0006886 | intracellular protein transport | -13.6397 | SCARB2,CHM,ATF2,KPNB1,MAN1A1,RAB5B,SEL1L,SRP54,TP53,XPO1,STX7,USO1,COPB2,SEC24C,GCC2,TRAM2,PREB,SEC23B,APPBP2,IPO8,AHCYL1,SEC24A,SEC23IP,SYNRG,SEC31A,XPO7,AP3M1,VPS41,ARFIP1,PIK3R4,IPO11,WDR11,VPS35,NUP107,ZDHHC6,SPG11,SYVN1,PIKFYVE,ATRX,BICD1,CD81,MSH2,RB1,GBF1,RABEP1,KNTC1,PIBF1,MIS12,RPF2,HOOK3,CHAMP1 |
| GO:0006259 | DNA metabolic process | -12.5574 | ATM,ATRX,CDC6,CYP1B1,ERCC5,GTF2H1,MSH2,ORC2,POLH,PRIM1,RBBP4,RFC1,TERF1,TOP1,TOP2A,TOP2B,TP53,SMC1A,CUL4A,RAD50,ASCC3,UBR2,UFL1,DICER1,ORC3,SAMHD1,RIF1,SMARCAD1,TAOK1,USP28,ZDBF2,NOC3L,METTL4,BRCC3,SMC6,CTC1,EPC1,FBH1,PRIMPOL,BCL2L1,ATF2,ASH2L,ZBTB40,CBX5,ZBTB38 |
| R-HSA-69278 | Cell Cycle, Mitotic | -12.3283 | CDC6,KPNB1,LBR,ORC2,PRIM1,RB1,RBBP4,RFC1,TOP2A,TP53,XPO1,AAAS,SMC1A,BLZF1,USO1,CDC23,CDC16,BTRC,KNTC1,CKAP5,NCAPD2,AKT3,ORC3,NSL1,ANAPC7,NUP107,TAOK1,MIS12,NUP37,BORA,CABLES1,NEK7,NUP43 |
| GO:0033044 | regulation of chromosome organization | -11.7535 | ATM,ATRX,CDC6,PARN,RB1,TERF1,TOP2A,CDC23,CDC16,KNTC1,NCAPD2,RAD50,ANAPC7,PBRM1,RESF1,NAT10,HMBOX1,SMC6,CTC1,SETDB2,NEK7,DHX36,APAF1,CYP1B1,MSH2,POLH,TP53,DEK,CUL4A,SF3B3,ORC3,RIF1,SMARCAD1,METTL4,BRCC3,OBI1,EPC1,FBH1,CCDC117,ZBTB38,BNIP3,PIP4K2A,MAPK9,ROCK2,CKAP5,FCHSD2,CNOT1,VPS35,ZDHHC6 |
| GO:0048193 | Golgi vesicle transport | -11.5115 | EPS15,GOLGA4,MYO5A,BLZF1,USO1,GBF1,RABEP1,COPB2,TRIP11,PREPL,SEC24C,GCC2,GOLGA5,PREB,SEC23B,SEC24A,SEC23IP,SEC31A,GOLPH3L,EXOC1,GOPC,TRAPPC11,TRAPPC6B,BICD1,SCARB2,CHM,MAN1A1,PIK3C2A,RAB5B,USP6NL,AKT3,STAM2,RAB30,VTA1,DENND4C,ACTR10,RALGAPB,DENND5B,FUT8,GFPT1,B4GALT1,PGM3,SEL1L,MLEC,MANEA,EDEM3,SYVN1,ALG11,AP3M1,CREB1,KPNB1,PIK3R4,HSD17B4,LBR,SLC27A2,OSBPL3,CHD9 |
| GO:0051052 | regulation of DNA metabolic process | -11.398 | APAF1,ATM,ATRX,CDC6,CYP1B1,MSH2,PARN,POLH,TERF1,TP53,DEK,CUL4A,RAD50,SF3B3,ORC3,RIF1,PBRM1,NAT10,SMARCAD1,METTL4,BRCC3,OBI1,HMBOX1,SMC6,CTC1,EPC1,FBH1,NEK7,CCDC117,DHX36,ZBTB38 |
| GO:0016050 | vesicle organization | -11.3333 | ATP6V0A1,LYST,CREB1,EPS15,PIP4K2A,RAB5B,STX7,USO1,GBF1,SEC24C,PREB,STAM2,SEC23B,SEC24A,SEC31A,AP3M1,VPS41,VTA1,GOLPH3L,TRAPPC11,SPG11,HOOK3,TRAPPC6B,PIKFYVE,CD9,RABEP1,SCARB2,TMEM106B |
| R-HSA-199991 | Membrane Trafficking | -10.8936 | BICD1,SCARB2,CHM,EPS15,GOLGA4,MAN1A1,MYO5A,PIK3C2A,RAB5B,USO1,GBF1,RABEP1,COPB2,TRIP11,SEC24C,GCC2,USP6NL,GOLGA5,AKT3,PREB,STAM2,SEC24A,SEC23IP,SEC31A,RAB30,VTA1,DENND4C,EXOC1,ACTR10,RALGAPB,TRAPPC11,TRAPPC6B,DENND5B |
| R-HSA-5653656 | Vesicle-mediated transport | -10.2128 | BICD1,SCARB2,CHM,EPS15,GOLGA4,MAN1A1,MYO5A,PIK3C2A,RAB5B,USO1,GBF1,RABEP1,COPB2,TRIP11,SEC24C,GCC2,USP6NL,GOLGA5,AKT3,PREB,STAM2,SEC24A,SEC23IP,SEC31A,RAB30,VTA1,DENND4C,EXOC1,ACTR10,RALGAPB,TRAPPC11,TRAPPC6B,DENND5B |
| R-HSA-446203 | Asparagine N-linked glycosylation | -9.28169 | FUT8,GFPT1,B4GALT1,MAN1A1,PGM3,SEL1L,USO1,GBF1,COPB2,SEC24C,MLEC,PREB,SEC24A,SEC23IP,SEC31A,ACTR10,MANEA,EDEM3,SYVN1,TRAPPC6B,ALG11 |
| GO:0000278 | mitotic cell cycle | -8.80914 | ATM,CDC6,ATF2,KPNB1,MSH2,RASA1,RB1,TP53,AAAS,SMC1A,CUL4A,CDC23,CDC16,ROCK2,KNTC1,CKAP5,NCAPD2,RAD50,PIBF1,NSL1,TUBD1,ANAPC7,PBRM1,EXOC1,TAOK1,MIS12,BRCC3,SETDB2,CHAMP1,CREB1,MAPK9,APAF1,RFC1,ATRX,BCL2L1,TERF1,XPO1,PIK3R4,NAT10,USP28,CEP97,BORA,CTC1,RIF1,STAT5B,GBF1,NEK7,LBR,IDE,CHM,ZNF420,EGR1,SOS1,WDR11,SESN3,GTF2H1,POLH,KDM4A,MGA,RBBP4,BTRC,AKT3,SMC6,PIK3C2A,AARS1,GCLC,ORC2,ORC3,PAK2,VPS35 |
| GO:0033365 | protein localization to organelle | -8.79858 | ATRX,BICD1,SCARB2,CD81,ATF2,KPNB1,MAN1A1,MSH2,RB1,SRP54,TP53,XPO1,GBF1,RABEP1,GCC2,TRAM2,KNTC1,PIBF1,IPO8,AP3M1,VPS41,PIK3R4,IPO11,VPS35,NUP107,MIS12,SPG11,RPF2,HOOK3,PIKFYVE,CHAMP1 |
| GO:1903047 | mitotic cell cycle process | -8.43117 | ATM,CDC6,ATF2,KPNB1,MSH2,RASA1,RB1,TP53,AAAS,SMC1A,CUL4A,CDC23,CDC16,ROCK2,KNTC1,CKAP5,NCAPD2,RAD50,PIBF1,NSL1,ANAPC7,EXOC1,TAOK1,MIS12,BRCC3,CHAMP1 |
| GO:0006974 | cellular response to DNA damage stimulus | -8.20478 | ATM,ATRX,BCL2L1,ATF2,ERCC5,GTF2H1,MSH2,POLH,RFC1,TOP2A,TP53,SMC1A,CUL4A,ASH2L,ZBTB40,RAD50,ASCC3,UFL1,CBX5,SAMHD1,RIF1,SMARCAD1,TAOK1,USP28,BRCC3,SMC6,CTC1,EPC1,FBH1,PRIMPOL,ZBTB38 |
| R-HSA-69620 | Cell Cycle Checkpoints | -8.00554 | ATM,CDC6,ORC2,TP53,XPO1,CDC23,CDC16,KNTC1,CKAP5,RAD50,ORC3,NSL1,ANAPC7,NUP107,TAOK1,MIS12,NUP37,BRCC3,NUP43 |
| GO:0006888 | endoplasmic reticulum to Golgi vesicle-mediated transport | -7.97948 | USO1,GBF1,COPB2,TRIP11,SEC24C,PREB,SEC23B,SEC24A,SEC23IP,SEC31A,GOPC,TRAPPC11,TRAPPC6B |
| GO:0051301 | cell division | -7.97667 | CDC6,EPB41,RASA1,RB1,TERF1,TOP1,TOP2A,SMC1A,CDC23,CDC16,ROCK2,KNTC1,CKAP5,NCAPD2,NSL1,ANAPC7,MIS18BP1,EXOC1,MIS12,NUP37,BRCC3,BORA,SETDB2,CABLES1,NUP43 |
| GO:0010638 | positive regulation of organelle organization | -7.97667 | ATM,ATRX,BNIP3,PARN,PIP4K2A,MAPK9,RB1,TERF1,TP53,CDC23,CDC16,ROCK2,CKAP5,FCHSD2,NCAPD2,RAD50,CNOT1,ANAPC7,RESF1,VPS35,ZDHHC6,HMBOX1,SETDB2,NEK7,DHX36 |
| R-HSA-69618 | Mitotic Spindle Checkpoint | -7.59203 | XPO1,CDC23,CDC16,KNTC1,CKAP5,NSL1,ANAPC7,NUP107,TAOK1,MIS12,NUP37,NUP43 |
| GO:0032204 | regulation of telomere maintenance | -7.16807 | ATM,ATRX,PARN,TERF1,RAD50,NAT10,HMBOX1,SMC6,CTC1,NEK7,DHX36 |
| GO:0006913 | nucleocytoplasmic transport | -7.10105 | ATF2,KPNB1,TP53,XPO1,AAAS,NEMF,IPO8,AHCYL1,XPO7,IPO11,IWS1,NUP107,THOC2,NUP37,SPG11,NUP43 |
| GO:0051169 | nuclear transport | -7.10105 | ATF2,KPNB1,TP53,XPO1,AAAS,NEMF,IPO8,AHCYL1,XPO7,IPO11,IWS1,NUP107,THOC2,NUP37,SPG11,NUP43 |
| GO:0006281 | DNA repair | -7.01599 | ATM,ATRX,ERCC5,GTF2H1,MSH2,POLH,RFC1,TP53,SMC1A,CUL4A,RAD50,ASCC3,UFL1,SAMHD1,RIF1,SMARCAD1,TAOK1,USP28,BRCC3,SMC6,EPC1,FBH1,PRIMPOL |
| R-HSA-3108232 | SUMO E3 ligases SUMOylate target proteins | -6.96049 | HDAC2,TOP1,TOP2A,TOP2B,TP53,AAAS,SMC1A,CASP8AP2,DDX17,CBX5,NUP107,NUP37,SMC6,NUP43 |
| GO:2001252 | positive regulation of chromosome organization | -6.94758 | ATM,ATRX,PARN,TERF1,NCAPD2,RAD50,RESF1,HMBOX1,SETDB2,NEK7,DHX36 |
| R-HSA-948021 | Transport to the Golgi and subsequent modification | -6.87243 | FUT8,B4GALT1,MAN1A1,USO1,GBF1,COPB2,SEC24C,PREB,SEC24A,SEC23IP,SEC31A,ACTR10,MANEA,TRAPPC6B |
| R-HSA-68886 | M Phase | -6.86917 | KPNB1,LBR,RB1,XPO1,AAAS,SMC1A,BLZF1,USO1,CDC23,CDC16,KNTC1,CKAP5,NCAPD2,NSL1,ANAPC7,NUP107,TAOK1,MIS12,NUP37,NEK7,NUP43 |
| R-HSA-2990846 | SUMOylation | -6.78608 | HDAC2,TOP1,TOP2A,TOP2B,TP53,AAAS,SMC1A,CASP8AP2,DDX17,CBX5,NUP107,NUP37,SMC6,NUP43 |
| R-HSA-68882 | Mitotic Anaphase | -6.33498 | KPNB1,LBR,XPO1,SMC1A,CDC23,CDC16,KNTC1,CKAP5,NSL1,ANAPC7,NUP107,TAOK1,MIS12,NUP37,NUP43 |
| R-HSA-2555396 | Mitotic Metaphase and Anaphase | -6.31161 | KPNB1,LBR,XPO1,SMC1A,CDC23,CDC16,KNTC1,CKAP5,NSL1,ANAPC7,NUP107,TAOK1,MIS12,NUP37,NUP43 |
| GO:0006900 | vesicle budding from membrane | -6.19656 | GBF1,SEC24C,PREB,SEC23B,SEC24A,SEC31A,GOLPH3L,TRAPPC11,TRAPPC6B |
| WP2516 | ATM signaling pathway | -6.15707 | ATM,CREB1,ATF2,MAPK9,TP53,SMC1A,RAD50 |
| GO:1904356 | regulation of telomere maintenance via telomere lengthening | -5.95781 | ATM,PARN,TERF1,NAT10,HMBOX1,CTC1,NEK7,DHX36 |
| R-HSA-2467813 | Separation of Sister Chromatids | -5.90086 | XPO1,SMC1A,CDC23,CDC16,KNTC1,CKAP5,NSL1,ANAPC7,NUP107,TAOK1,MIS12,NUP37,NUP43 |
| hsa03013 | Nucleocytoplasmic transport | -5.86926 | KPNB1,XPO1,AAAS,IPO8,XPO7,IPO11,NUP107,THOC2,NUP37,NUP43 |
| R-HSA-4615885 | SUMOylation of DNA replication proteins | -5.79465 | TOP1,TOP2A,TOP2B,AAAS,NUP107,NUP37,NUP43 |
| GO:0051054 | positive regulation of DNA metabolic process | -5.74434 | ATM,ATRX,CYP1B1,MSH2,PARN,TERF1,RAD50,RIF1,PBRM1,BRCC3,HMBOX1,CTC1,EPC1,NEK7,CCDC117,DHX36 |
| GO:0032206 | positive regulation of telomere maintenance | -5.6919 | ATM,ATRX,PARN,TERF1,RAD50,HMBOX1,NEK7,DHX36 |
| R-HSA-9648025 | EML4 and NUDC in mitotic spindle formation | -5.55116 | XPO1,KNTC1,CKAP5,NSL1,NUP107,TAOK1,MIS12,NUP37,NEK7,NUP43 |
| WP707 | DNA damage response | -5.54332 | APAF1,ATM,CREB1,RB1,RFC1,TP53,SMC1A,RAD50 |
| GO:0006403 | RNA localization | -5.44939 | ATM,BICD1,KPNB1,ATXN2,XPO1,AAAS,CKAP5,IWS1,NUP107,THOC2,NUP37,NUP43 |
| R-HSA-141424 | Amplification of signal from the kinetochores | -5.38431 | XPO1,KNTC1,CKAP5,NSL1,NUP107,TAOK1,MIS12,NUP37,NUP43 |
| R-HSA-141444 | Amplification of signal from unattached kinetochores via a MAD2 inhibitory signal | -5.38431 | XPO1,KNTC1,CKAP5,NSL1,NUP107,TAOK1,MIS12,NUP37,NUP43 |
| GO:0032210 | regulation of telomere maintenance via telomerase | -5.30182 | ATM,PARN,TERF1,NAT10,HMBOX1,CTC1,NEK7 |
| R-HSA-2500257 | Resolution of Sister Chromatid Cohesion | -5.26098 | XPO1,SMC1A,KNTC1,CKAP5,NSL1,NUP107,TAOK1,MIS12,NUP37,NUP43 |
| R-HSA-177243 | Interactions of Rev with host cellular proteins | -5.22203 | KPNB1,XPO1,AAAS,NUP107,NUP37,NUP43 |
| GO:0051236 | establishment of RNA localization | -5.09907 | ATM,KPNB1,ATXN2,XPO1,AAAS,CKAP5,IWS1,NUP107,THOC2,NUP37,NUP43 |
| R-HSA-162906 | HIV Infection | -4.99483 | GTF2A1,GTF2H1,KPNB1,PAK2,TBP,XPO1,AAAS,BTRC,PSIP1,VTA1,NUP107,NUP37,NUP43 |
| GO:0090114 | COPII-coated vesicle budding | -4.9462 | SEC24C,PREB,SEC23B,SEC24A,SEC31A,TRAPPC11 |
| R-HSA-68877 | Mitotic Prometaphase | -4.84656 | XPO1,SMC1A,KNTC1,CKAP5,NCAPD2,NSL1,NUP107,TAOK1,MIS12,NUP37,NEK7,NUP43 |
| GO:2000278 | regulation of DNA biosynthetic process | -4.62369 | ATM,CYP1B1,PARN,TERF1,TP53,NAT10,HMBOX1,CTC1,NEK7 |
| R-HSA-162587 | HIV Life Cycle | -4.62064 | GTF2A1,GTF2H1,TBP,XPO1,AAAS,PSIP1,VTA1,NUP107,NUP37,NUP43 |
| GO:0090110 | COPII-coated vesicle cargo loading | -4.61952 | SEC24C,SEC23B,SEC24A,SEC31A |
| R-HSA-195258 | RHO GTPase Effectors | -4.611 | KTN1,PAK2,XPO1,ROCK2,KNTC1,CKAP5,NSL1,CYFIP2,PIK3R4,GOPC,NUP107,TAOK1,MIS12,NUP37,NUP43 |
| GO:0072594 | establishment of protein localization to organelle | -4.59567 | SCARB2,ATF2,KPNB1,MAN1A1,SRP54,TP53,GCC2,TRAM2,IPO8,AP3M1,VPS41,PIK3R4,IPO11,NUP107,SPG11 |
| R-HSA-204005 | COPII-mediated vesicle transport | -4.57476 | USO1,SEC24C,PREB,SEC24A,SEC23IP,SEC31A,TRAPPC6B |
| R-HSA-199977 | ER to Golgi Anterograde Transport | -4.49737 | USO1,GBF1,COPB2,SEC24C,PREB,SEC24A,SEC23IP,SEC31A,ACTR10,TRAPPC6B |
| GO:0050657 | nucleic acid transport | -4.37895 | KPNB1,ATXN2,XPO1,AAAS,CKAP5,IWS1,NUP107,THOC2,NUP37,NUP43 |
| GO:0050658 | RNA transport | -4.37895 | KPNB1,ATXN2,XPO1,AAAS,CKAP5,IWS1,NUP107,THOC2,NUP37,NUP43 |
| R-HSA-168271 | Transport of Ribonucleoproteins into the Host Nucleus | -4.3615 | KPNB1,AAAS,NUP107,NUP37,NUP43 |
| R-HSA-168333 | NEP/NS2 Interacts with the Cellular Export Machinery | -4.3615 | XPO1,AAAS,NUP107,NUP37,NUP43 |
| R-HSA-162909 | Host Interactions of HIV factors | -4.32 | KPNB1,PAK2,XPO1,AAAS,BTRC,PSIP1,NUP107,NUP37,NUP43 |
| R-HSA-168274 | Export of Viral Ribonucleoproteins from Nucleus | -4.22549 | XPO1,AAAS,NUP107,NUP37,NUP43 |
| R-HSA-180746 | Nuclear import of Rev protein | -4.22549 | KPNB1,AAAS,NUP107,NUP37,NUP43 |
| R-HSA-180910 | Vpr-mediated nuclear import of PICs | -4.22549 | AAAS,PSIP1,NUP107,NUP37,NUP43 |
| GO:0035459 | vesicle cargo loading | -4.22549 | SEC24C,SEC23B,SEC24A,SEC31A,AP3M1 |
| GO:0006302 | double-strand break repair | -4.20561 | ATM,ERCC5,MSH2,TP53,RAD50,SAMHD1,SMARCAD1,BRCC3,SMC6,EPC1,FBH1 |
| R-HSA-162599 | Late Phase of HIV Life Cycle | -4.16727 | GTF2A1,GTF2H1,TBP,XPO1,AAAS,VTA1,NUP107,NUP37,NUP43 |
| R-HSA-165054 | Rev-mediated nuclear export of HIV RNA | -4.16098 | XPO1,AAAS,NUP107,NUP37,NUP43 |
| R-HSA-1169410 | Antiviral mechanism by IFN-stimulated genes | -4.1164 | FLNB,KPNB1,RNASEL,AAAS,NUP107,NUP37,NUP43 |
| R-HSA-9679506 | SARS-CoV Infections | -4.10978 | FUT8,HDAC2,RB1,RBBP4,AAAS,ROCK2,SEC24C,AKT3,SEC24A,GEMIN5,VPS41,PIK3R4,NUP107,NUP37,NUP43 |
| R-HSA-3301854 | Nuclear Pore Complex (NPC) Disassembly | -4.09859 | AAAS,NUP107,NUP37,NEK7,NUP43 |
| R-HSA-5663220 | RHO GTPases Activate Formins | -4.06997 | XPO1,KNTC1,CKAP5,NSL1,NUP107,TAOK1,MIS12,NUP37,NUP43 |
| R-HSA-176033 | Interactions of Vpr with host cellular proteins | -4.03822 | AAAS,PSIP1,NUP107,NUP37,NUP43 |
| GO:1904358 | positive regulation of telomere maintenance via telomere lengthening | -3.97973 | ATM,PARN,HMBOX1,NEK7,DHX36 |
| GO:0034504 | protein localization to nucleus | -3.96811 | ATF2,KPNB1,TP53,XPO1,IPO8,IPO11,NUP107,SPG11,RPF2,PIKFYVE |
| GO:0015931 | nucleobase-containing compound transport | -3.94486 | KPNB1,ATXN2,XPO1,AAAS,SLC33A1,CKAP5,IWS1,NUP107,THOC2,NUP37,NUP43 |
| R-HSA-168276 | NS1 Mediated Effects on Host Pathways | -3.81456 | KPNB1,AAAS,NUP107,NUP37,NUP43 |
| CORUM:87 | Nup 107-160 subcomplex | -3.78079 | NUP107,NUP37,NUP43 |
| R-HSA-159231 | Transport of Mature mRNA Derived from an Intronless Transcript | -3.76263 | AAAS,CPSF2,NUP107,NUP37,NUP43 |
| R-HSA-159234 | Transport of Mature mRNAs Derived from Intronless Transcripts | -3.71214 | AAAS,CPSF2,NUP107,NUP37,NUP43 |
| GO:0006310 | DNA recombination | -3.6404 | ATM,ERCC5,MSH2,TOP2A,TOP2B,RAD50,UBR2,SAMHD1,SMC6,EPC1,FBH1 |
| R-HSA-9705683 | SARS-CoV-2-host interactions | -3.52254 | AAAS,SEC24C,AKT3,SEC24A,GEMIN5,VPS41,PIK3R4,NUP107,NUP37,NUP43 |
| R-HSA-4551638 | SUMOylation of chromatin organization proteins | -3.51923 | HDAC2,AAAS,CBX5,NUP107,NUP37,NUP43 |
| R-HSA-1169408 | ISG15 antiviral mechanism | -3.4861 | FLNB,KPNB1,AAAS,NUP107,NUP37,NUP43 |
| GO:0051972 | regulation of telomerase activity | -3.47885 | PARN,TERF1,TP53,HMBOX1,NEK7 |
| R-HSA-9694516 | SARS-CoV-2 Infection | -3.46342 | FUT8,RB1,AAAS,SEC24C,AKT3,SEC24A,GEMIN5,VPS41,PIK3R4,NUP107,NUP37,NUP43 |
| WP1982 | Sterol regulatory element-binding proteins (SREBP) signaling | -3.45351 | CREB1,KPNB1,SEC24C,SEC23B,SEC24A,SEC31A |
| R-HSA-9615933 | Postmitotic nuclear pore complex (NPC) reformation | -3.43288 | KPNB1,NUP107,NUP37,NUP43 |
| R-HSA-3108214 | SUMOylation of DNA damage response and repair proteins | -3.32824 | AAAS,SMC1A,NUP107,NUP37,SMC6,NUP43 |
| R-HSA-5619115 | Disorders of transmembrane transporters | -3.32628 | SLC26A2,SEL1L,AAAS,SLC33A1,ERLIN1,ERLEC1,NUP107,NUP37,NUP43 |
| R-HSA-983170 | Antigen Presentation: Folding, assembly and peptide loading of class I MHC | -3.31025 | SEC24C,SEC24A,SEC31A,PIK3R4 |
| GO:0006606 | protein import into nucleus | -3.30578 | ATF2,KPNB1,TP53,IPO8,IPO11,NUP107,SPG11 |
| R-HSA-68875 | Mitotic Prophase | -3.29139 | RB1,AAAS,BLZF1,USO1,NUP107,NUP37,NEK7,NUP43 |
| R-HSA-2980766 | Nuclear Envelope Breakdown | -3.27257 | AAAS,NUP107,NUP37,NEK7,NUP43 |
| R-HSA-191859 | snRNP Assembly | -3.23408 | AAAS,GEMIN5,NUP107,NUP37,NUP43 |
| R-HSA-194441 | Metabolism of non-coding RNA | -3.23408 | AAAS,GEMIN5,NUP107,NUP37,NUP43 |
| GO:0051170 | import into nucleus | -3.21098 | ATF2,KPNB1,TP53,IPO8,IPO11,NUP107,SPG11 |
| R-HSA-170822 | Regulation of Glucokinase by Glucokinase Regulatory Protein | -3.19687 | AAAS,NUP107,NUP37,NUP43 |
| R-HSA-5619107 | Defective TPR may confer susceptibility towards thyroid papillary carcinoma (TPC) | -3.19687 | AAAS,NUP107,NUP37,NUP43 |
| R-HSA-72202 | Transport of Mature Transcript to Cytoplasm | -3.12664 | AAAS,CPSF2,NUP107,THOC2,NUP37,NUP43 |
| R-HSA-8957322 | Metabolism of steroids | -3.06002 | HSD17B4,KPNB1,LBR,SEC24C,SEC24A,SLC27A2,OSBPL3,CHD9 |
| R-HSA-159227 | Transport of the SLBP independent Mature mRNA | -3.04154 | AAAS,NUP107,NUP37,NUP43 |
| R-HSA-4085377 | SUMOylation of SUMOylation proteins | -3.04154 | AAAS,NUP107,NUP37,NUP43 |
| GO:0032212 | positive regulation of telomere maintenance via telomerase | -3.04154 | ATM,PARN,HMBOX1,NEK7 |
| GO:0061025 | membrane fusion | -3.00576 | CD9,PIP4K2A,STX7,USO1,RABEP1,VPS41,SPG11,PIKFYVE |
| R-HSA-159230 | Transport of the SLBP Dependant Mature mRNA | -2.99321 | AAAS,NUP107,NUP37,NUP43 |
| R-HSA-9705671 | SARS-CoV-2 activates/modulates innate and adaptive immune responses | -2.90958 | AAAS,SEC24C,SEC24A,PIK3R4,NUP107,NUP37,NUP43 |
| R-HSA-3232142 | SUMOylation of ubiquitinylation proteins | -2.85717 | AAAS,NUP107,NUP37,NUP43 |
| GO:0051028 | mRNA transport | -2.85044 | XPO1,AAAS,IWS1,NUP107,THOC2,NUP37,NUP43 |
| R-HSA-5619102 | SLC transporter disorders | -2.7783 | SLC26A2,AAAS,SLC33A1,NUP107,NUP37,NUP43 |
| GO:0006906 | vesicle fusion | -2.73352 | PIP4K2A,STX7,USO1,VPS41,SPG11,PIKFYVE |
| R-HSA-3371453 | Regulation of HSF1-mediated heat shock response | -2.71364 | ATM,AAAS,NUP107,NUP37,NUP43 |
| R-HSA-211000 | Gene Silencing by RNA | -2.68298 | AAAS,IPO8,DICER1,DROSHA,NUP107,NUP37,NUP43 |
| R-HSA-168325 | Viral Messenger RNA Synthesis | -2.65578 | AAAS,NUP107,NUP37,NUP43 |
| GO:0090174 | organelle membrane fusion | -2.64721 | PIP4K2A,STX7,USO1,VPS41,SPG11,PIKFYVE |
| GO:2000573 | positive regulation of DNA biosynthetic process | -2.60572 | ATM,CYP1B1,PARN,HMBOX1,NEK7 |
| R-HSA-159236 | Transport of Mature mRNA derived from an Intron-Containing Transcript | -2.55439 | AAAS,NUP107,THOC2,NUP37,NUP43 |
| R-HSA-2995410 | Nuclear Envelope (NE) Reassembly | -2.55439 | KPNB1,LBR,NUP107,NUP37,NUP43 |
| R-HSA-4570464 | SUMOylation of RNA binding proteins | -2.51324 | AAAS,NUP107,NUP37,NUP43 |
| GO:0000724 | double-strand break repair via homologous recombination | -2.48638 | ATM,ERCC5,SAMHD1,SMC6,EPC1,FBH1 |
| GO:0000725 | recombinational repair | -2.4113 | ATM,ERCC5,SAMHD1,SMC6,EPC1,FBH1 |
| R-HSA-168255 | Influenza Infection | -2.38685 | KPNB1,DNAJC3,XPO1,AAAS,NUP107,NUP37,NUP43 |
| GO:0044088 | regulation of vacuole organization | -2.38417 | SCARB2,PIP4K2A,TMEM106B,PIKFYVE |
| R-HSA-72203 | Processing of Capped Intron-Containing Pre-mRNA | -2.33974 | AAAS,AQR,SF3B3,SRRM2,CPSF2,NUP107,THOC2,NUP37,NUP43 |
| R-HSA-71387 | Metabolism of carbohydrates | -2.29784 | AGL,VCAN,SLC26A2,B4GALT1,PHKB,AAAS,CHST15,NUP107,NUP37,NUP43 |
| GO:0048284 | organelle fusion | -2.28752 | PIP4K2A,STX7,USO1,VPS41,SPG11,PIKFYVE |
| R-HSA-1655829 | Regulation of cholesterol biosynthesis by SREBP (SREBF) | -2.26646 | KPNB1,SEC24C,SEC24A,CHD9 |
| R-HSA-3371556 | Cellular response to heat stress | -2.23623 | ATM,AAAS,NUP107,NUP37,NUP43 |
| R-HSA-6784531 | tRNA processing in the nucleus | -2.21134 | AAAS,NUP107,NUP37,NUP43 |
| hsa05014 | Amyotrophic lateral sclerosis | -2.11075 | APAF1,BCL2L1,ATXN2,TP53,PIK3R4,ATG2B,ACTR10,NUP107,NUP37,SPG11,NUP43 |
| R-HSA-9609690 | HCMV Early Events | -2.09517 | CREB1,RBBP4,AAAS,NUP107,NUP37,NUP43 |
| R-HSA-5694530 | Cargo concentration in the ER | -2.06698 | SEC24C,PREB,SEC24A |
| GO:0051973 | positive regulation of telomerase activity | -2.03094 | PARN,HMBOX1,NEK7 |
