# Supplementary material for: N6-methyladenosine reader YTHDF2 promotes multiple myeloma cell proliferation through EGR1/p21cip1/waf1/CDK2-Cyclin E1 axis-mediated cell cycle transition
Source: Oncogene. 2023 Apr 3;42(20):1607–19. doi: 10.1038/s41388-023-02675-w (PMC10181929; doi:10.1038/s41388-023-02675-w)
Supplement: Supplementary file 2 — Supplementary materials_original WB [file 41388_2023_2675_MOESM2_ESM.pdf]

Figure 2B

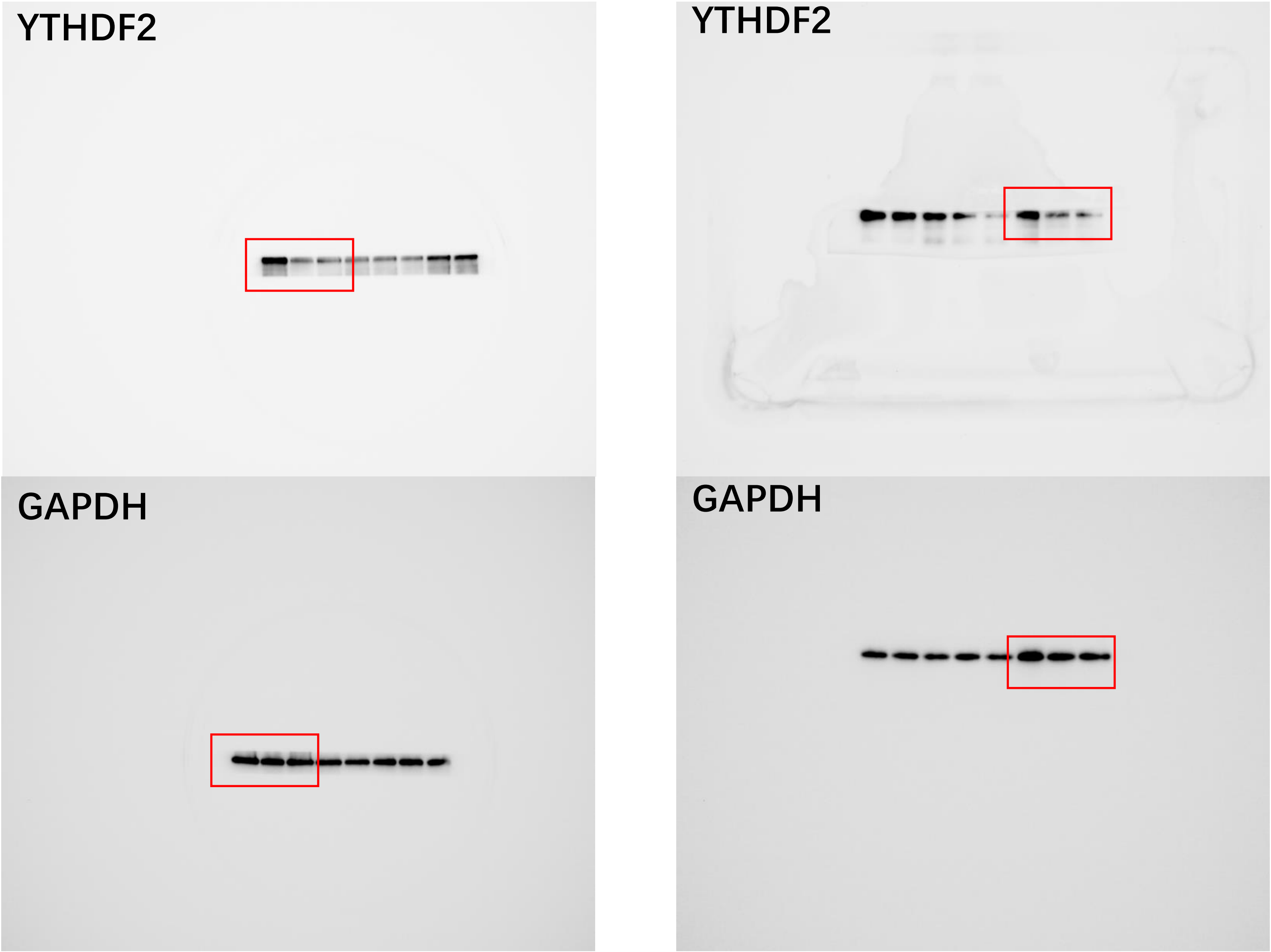

1. RPMI8226 siNC
2. RPMI8226 siYTHDF2-1
3. RPMI8226 siYTHDF2-2

1. NCIH929 siNC
2. NCIH929 siYTHDF2-1
3. NCIH929 siYTHDF2-2

Figure 3J

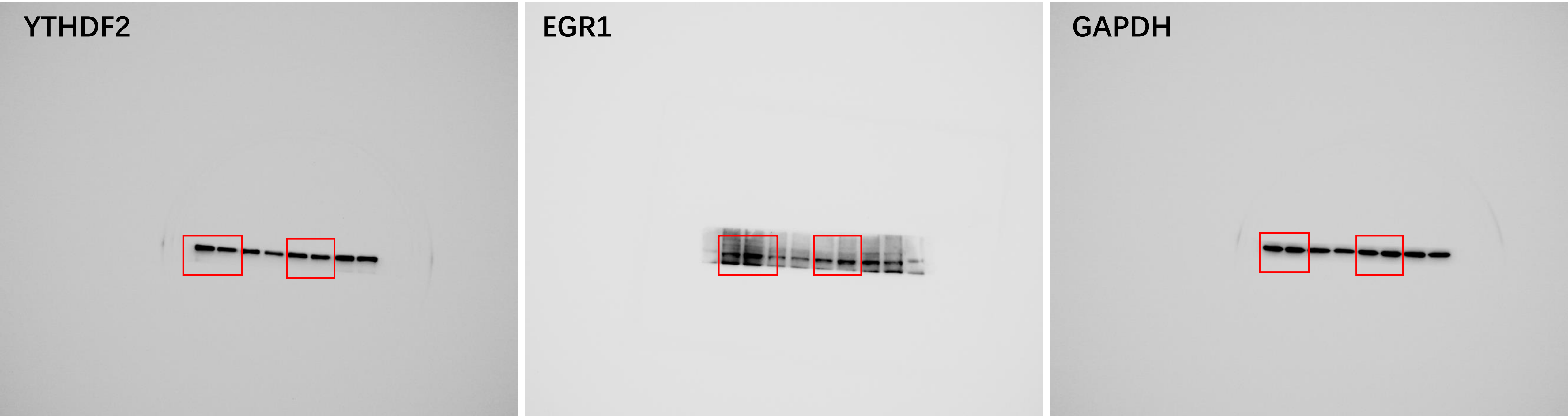

1. RPMI8226 siNC
2. RPMI8226 siYTHDF2
3. NCIH929 siNC
4. NCIH929 siYTHDF2

Figure 4B

1. RPMI8226 siNC
2. RPMI8226 siEGR1-1
3. RPMI8226 siEGR1-2

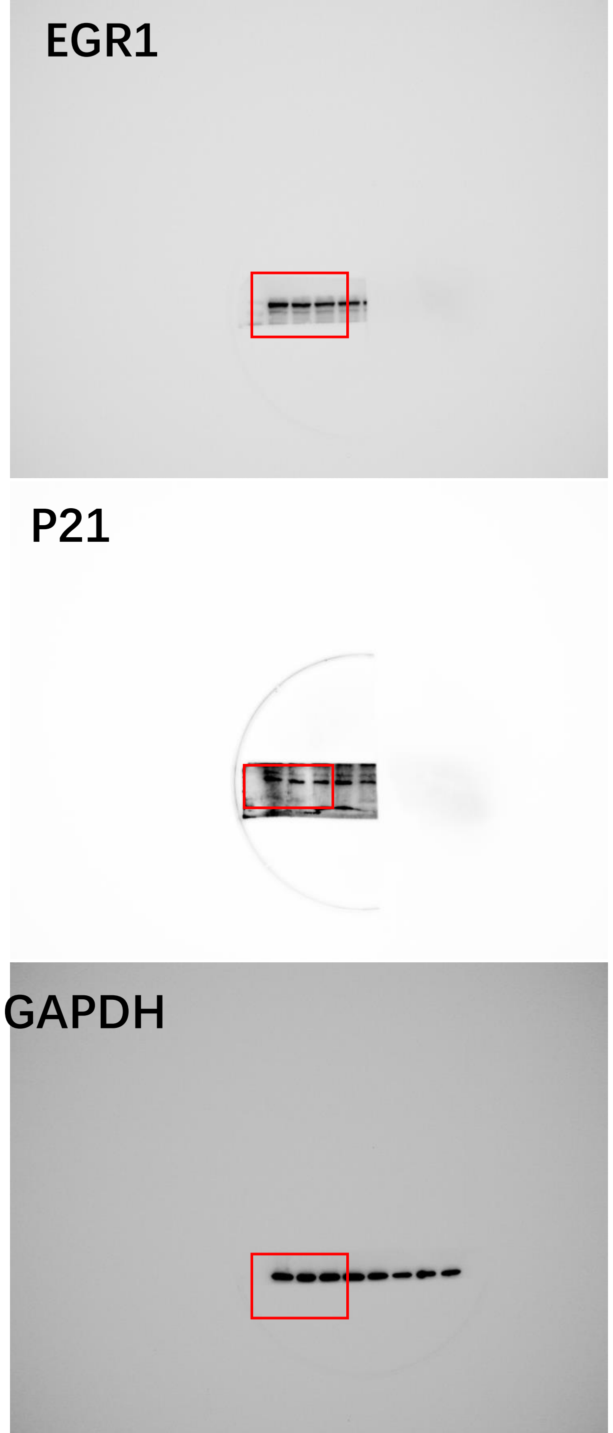

1. NCIH929 siNC
2. NCIH929 siEGR1

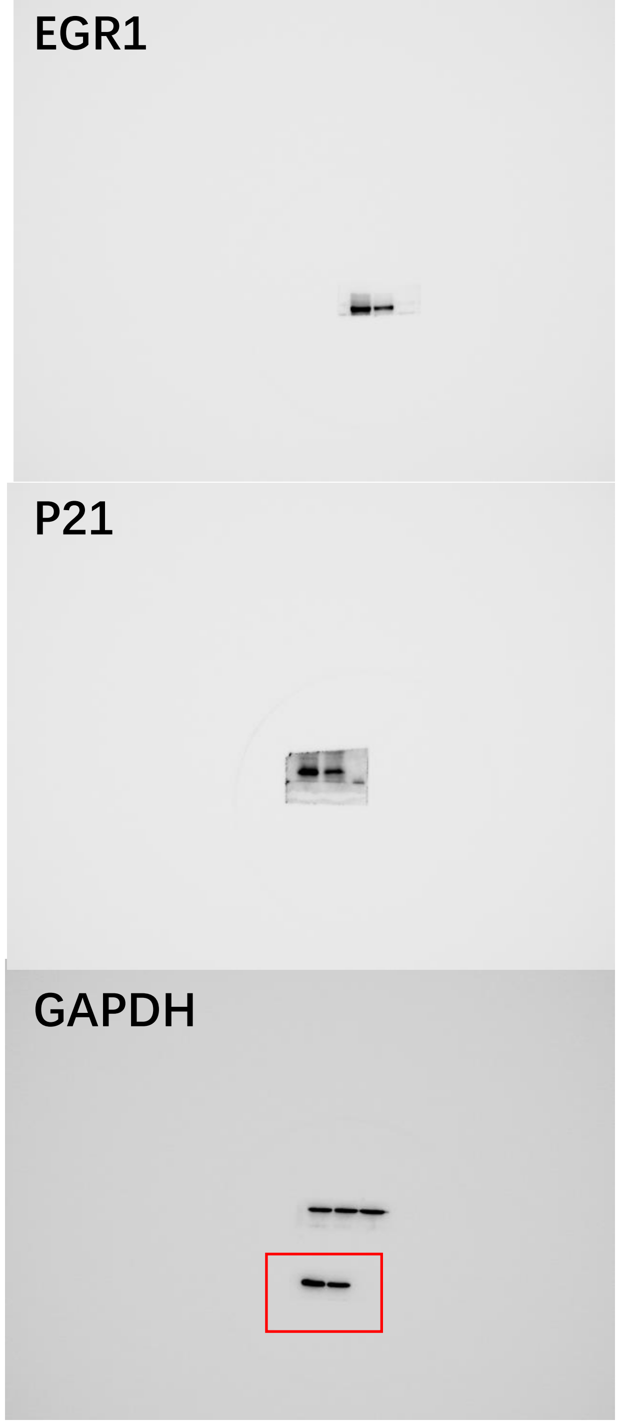

Figure 4I

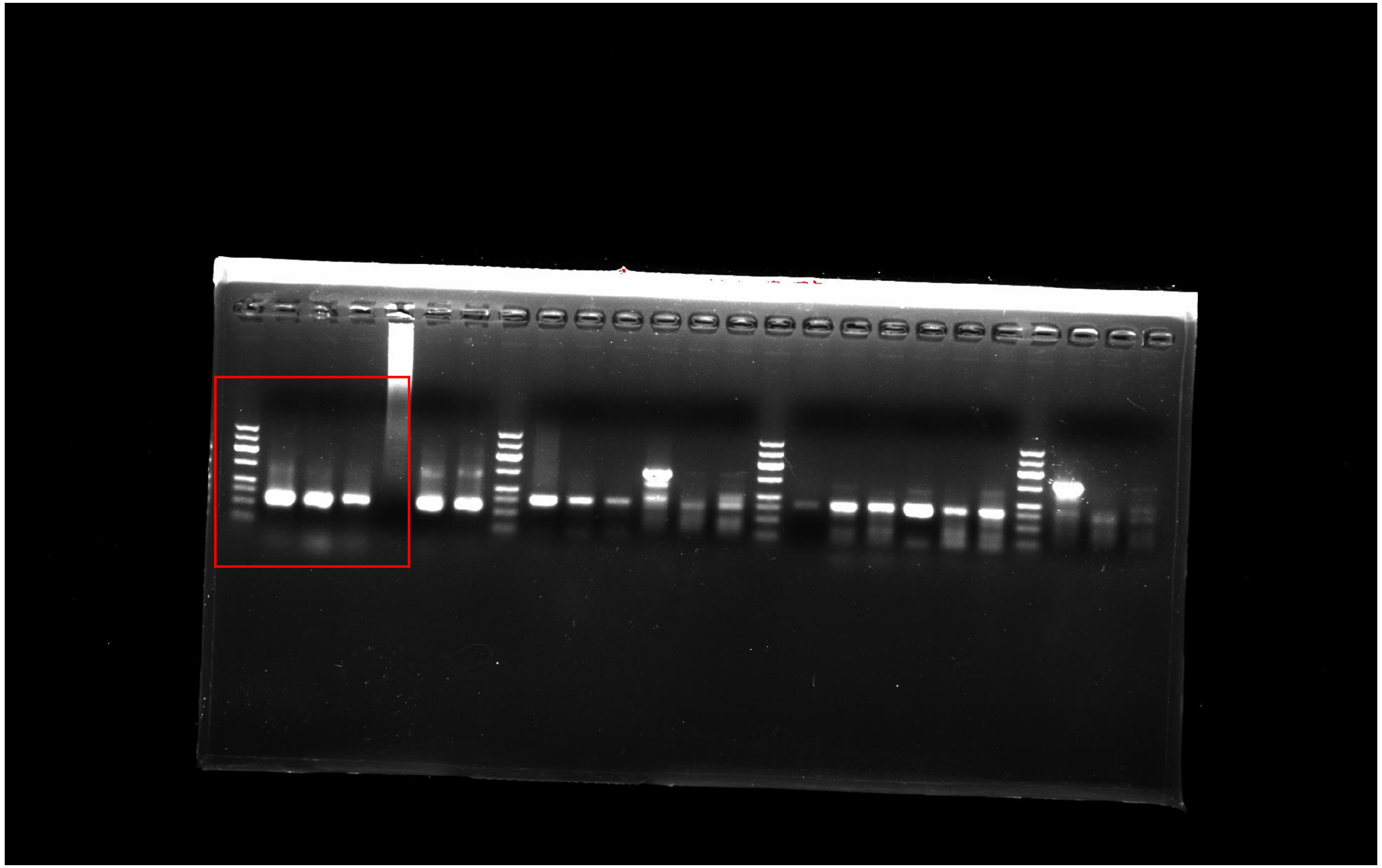

1. Input
2. IP
3. IgG

Figure 5A-1

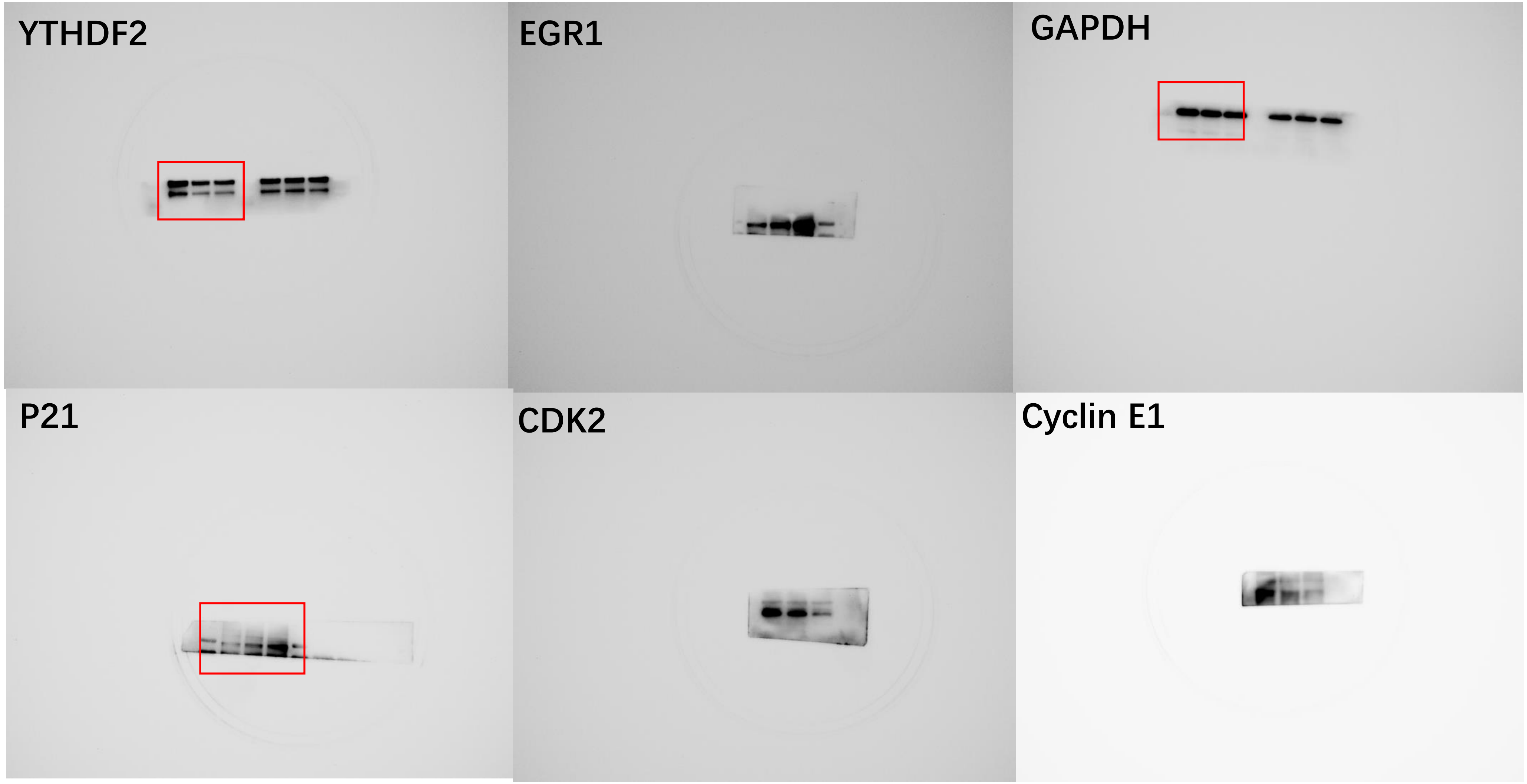

1. NCIH929 siNC
2. NCIH929 siYTHDF2-1
3. NCIH929 siYTHDF2-2

Figure 5A-2

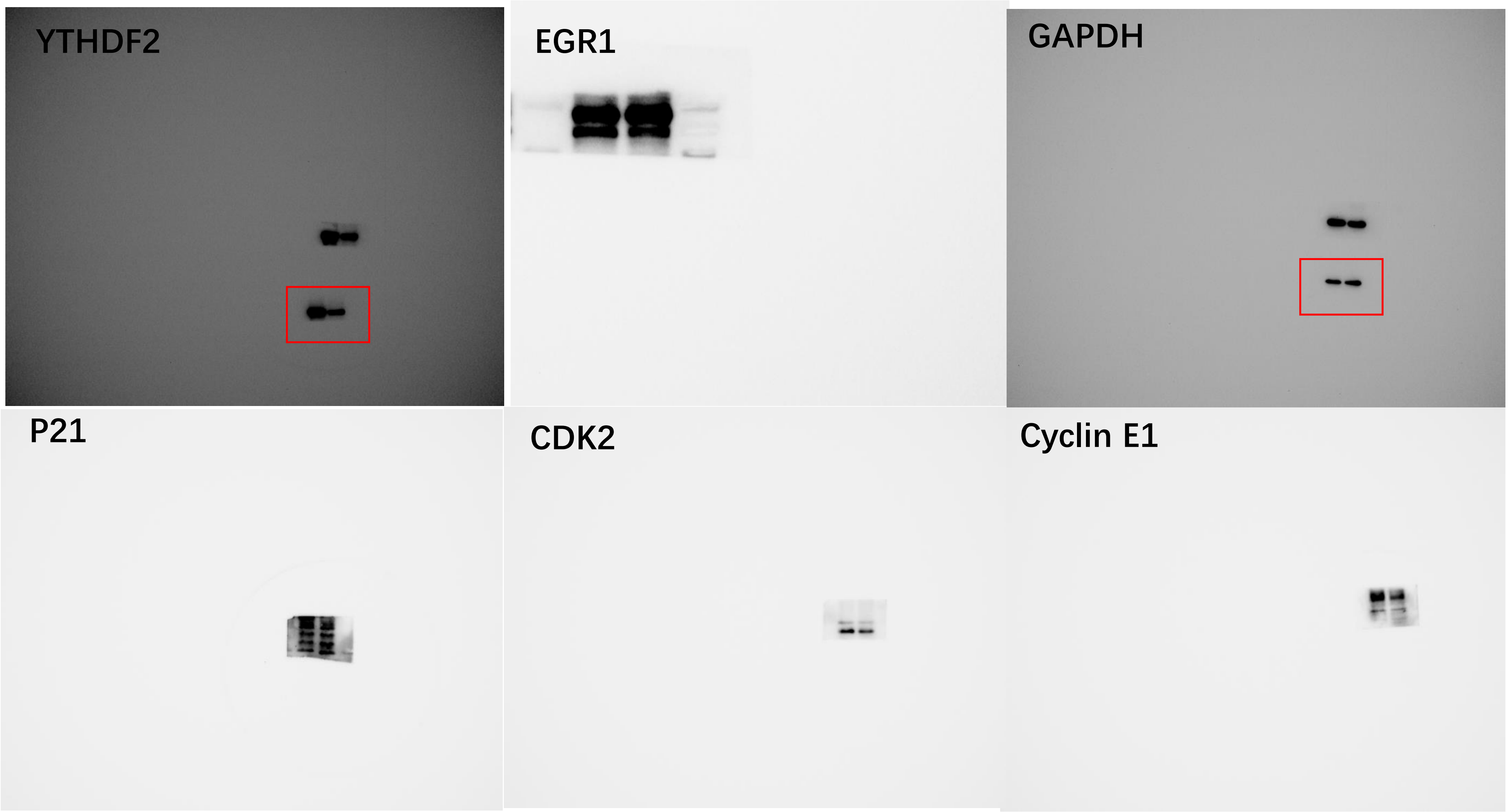

1. RPMI8226 siNC
2. RPMI8226 siYTHDF2

Figure 5B-1

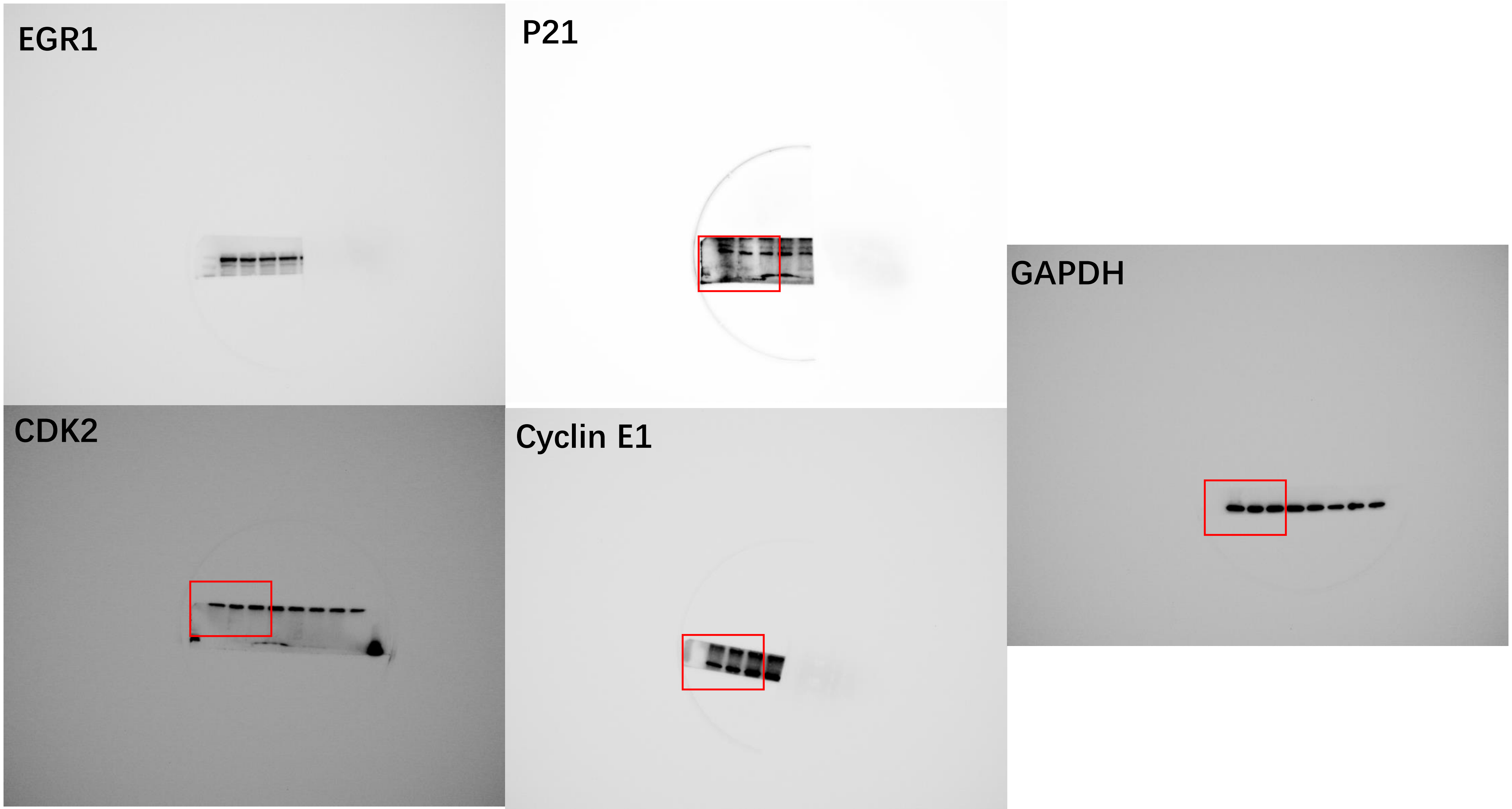

1. RPMI8226 siNC
2. RPMI8226 siEGR1-1
3. RPMI8226 siEGR1-2

Figure 5B-2

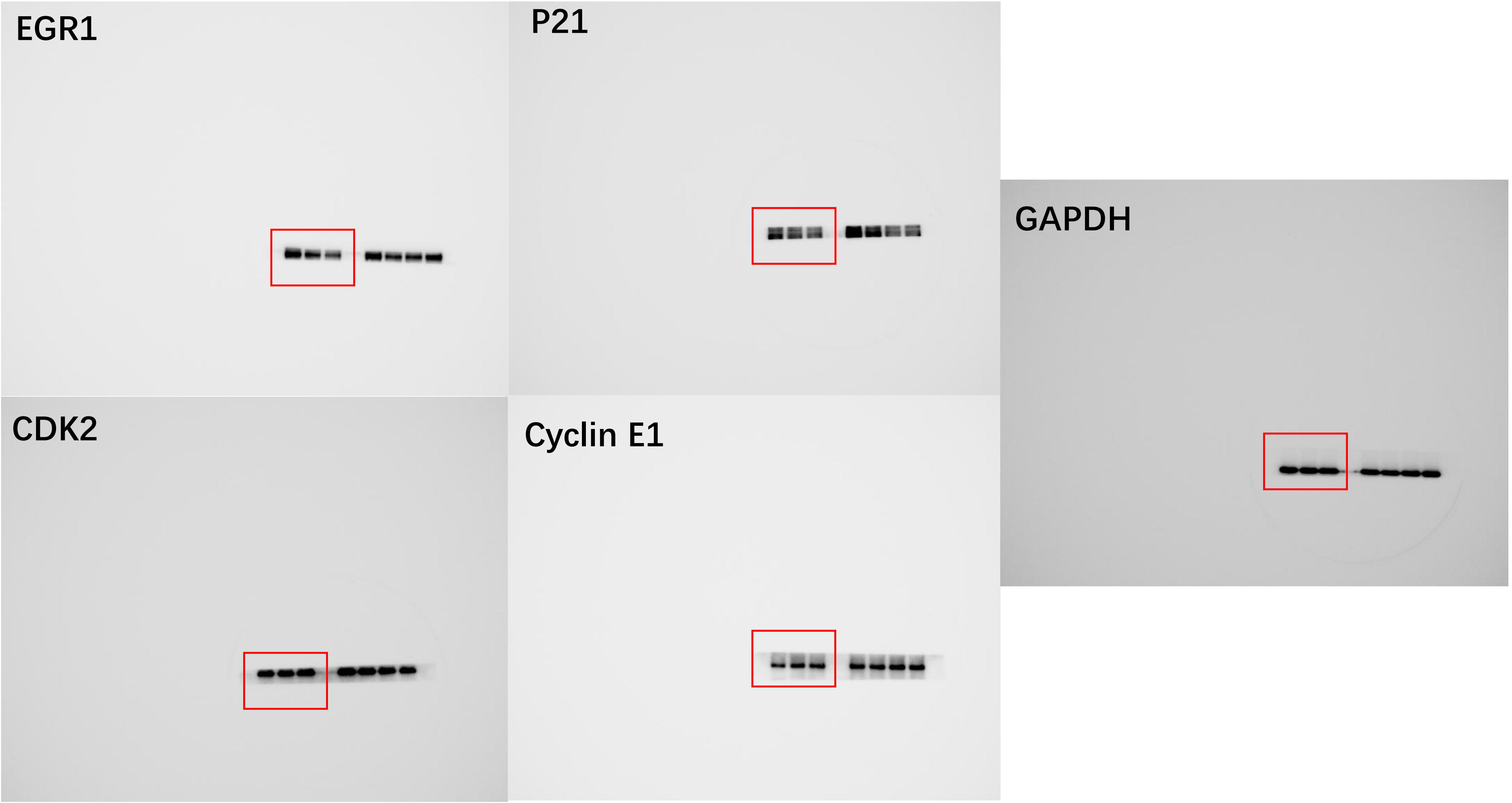

1. NCIH929 siNC
2. NCIH929 siEGR1-1
3. NCIH929 siEGR1-2

Figure 5E-1

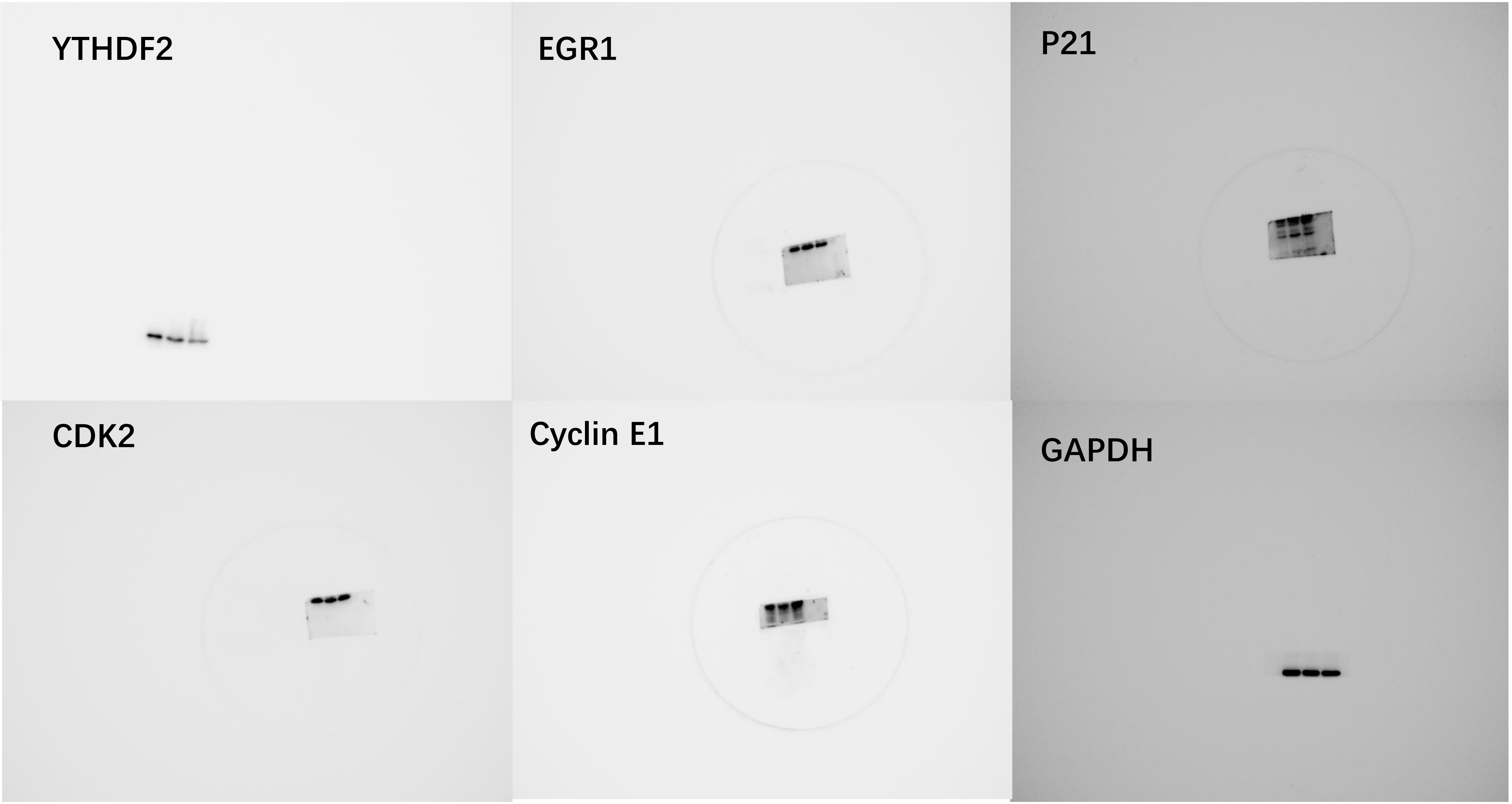

1. RPMI8226 siNC
2. RPMI8226 siYTHDF2
3. RPMI8226 siYTHDF2+siEGR1

Figure 5E-2

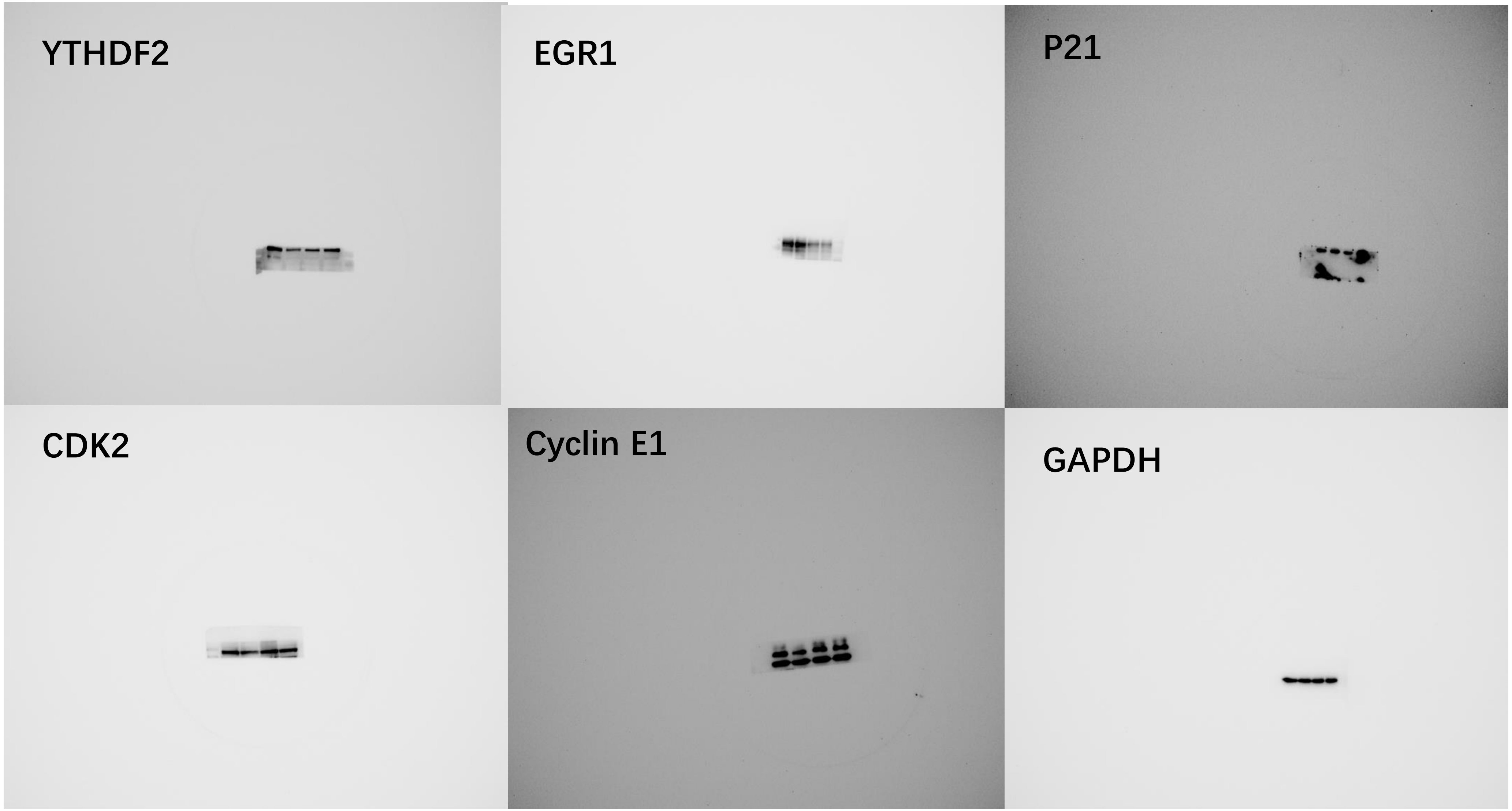

1. NCIH929 siNC
2. NCIH929 siYTHDF2
3. NCIH929 siYTHDF2+siEGR1#1
4. NCIH929 siYTHDF2+siEGR1#2

Figure 7B-1

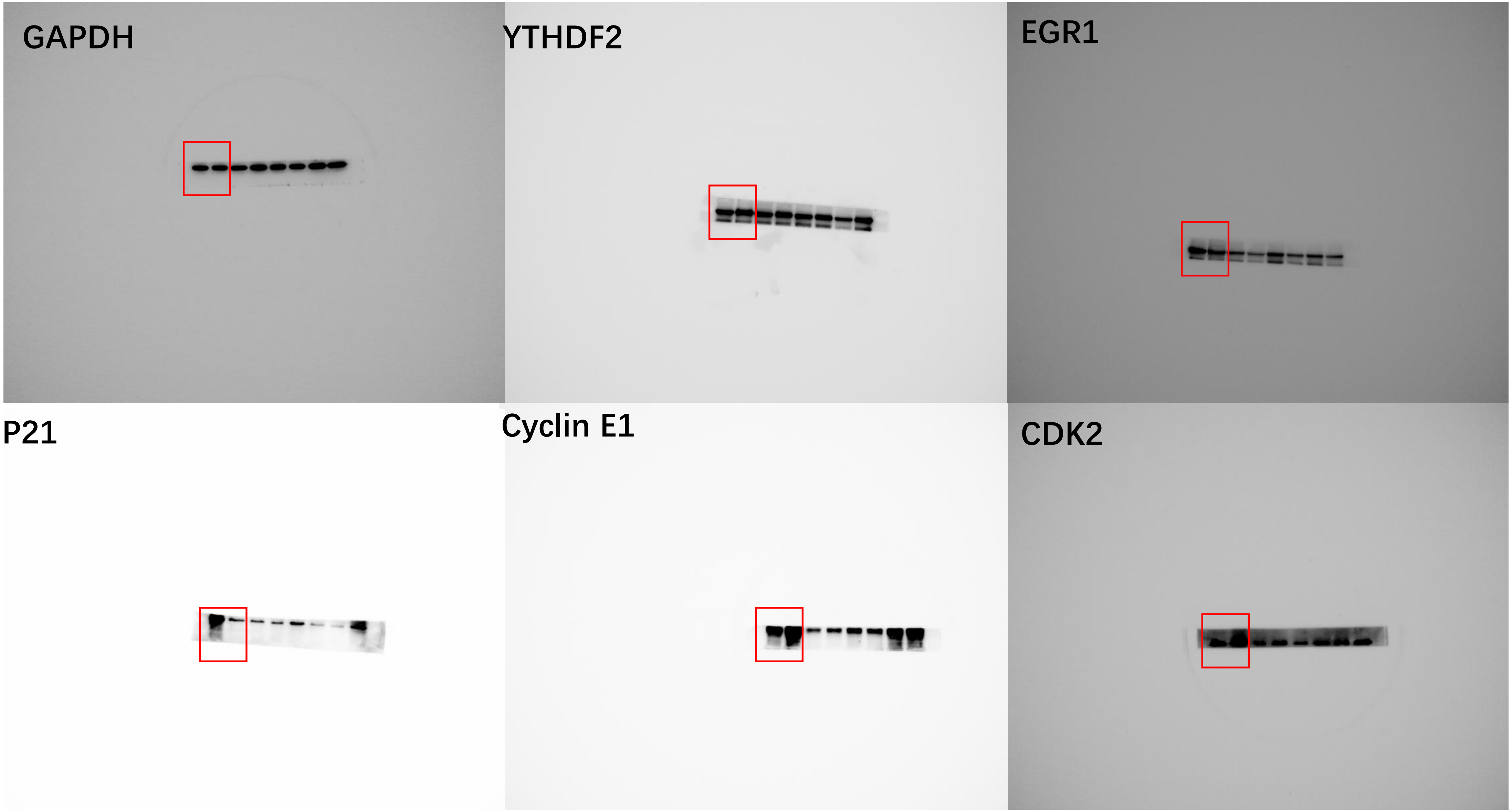

1. RPMI8226 LVNC
2. RPMI8226 LV-oeYTHDF2

Figure 7B-2

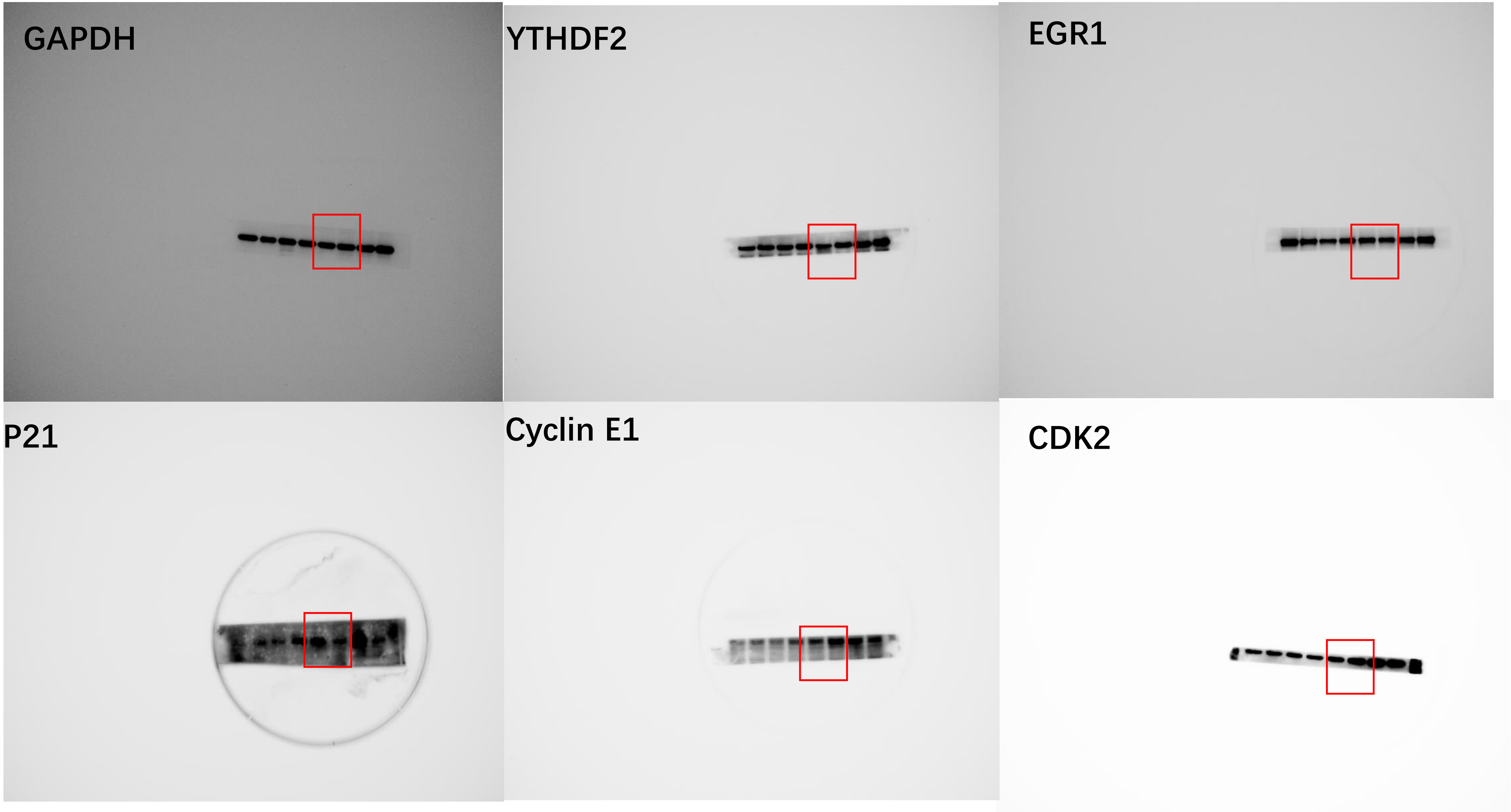

1. NCI-H929 LVNC
2. NCI-H929 LV-oeYTHDF2
